# Effects of the Hydrophilic–Lipophilic Balance of Alternating Peptides on Self-Assembly and Thermo-Responsive Behaviors

**DOI:** 10.3390/ijms20184604

**Published:** 2019-09-17

**Authors:** Abu Bin Ihsan, Mahmuda Nargis, Yasuhito Koyama

**Affiliations:** Department of Pharmaceutical Engineering, Faculty of Engineering, Toyama Prefectural University, 5180 Kurokawa, Imizu, Toyama 939-0398, Japan; ihsan@pu-toyama.ac.jp (A.B.I.); nargis@pu-toyama.ac.jp (M.N.)

**Keywords:** alternating peptide, amphiphilic peptide, multi-component polycondensation, cloud point, critical micelle concentration

## Abstract

A series of *N*-substituted poly(Gly–*alter*–Val) peptides were successfully synthesized for the systematic evaluation of the micellization behavior of alternating peptides. Three-component polymerization employing an aldehyde, a primary ammonium chloride, and potassium isocyanoacetate afforded four alternating peptides in excellent yields. We investigated the dependence of the hydrophilic–lipophilic balance of alternating peptides on the micellization behavior. All the aqueous solutions of alternating peptides exhibited upper critical solution temperature (UCST) behaviors, strongly indicating that the alternating binary pattern would mainly contribute to the UCST behaviors. The cloud points of alternating peptides shifted to higher temperatures as the side chains became more hydrophilic, which is opposite to the trend of typical surfactants. Such unusual micellization behaviors appeared to be dependent on the quasi-stable structure of single polymer chains formed in water.

## 1. Introduction

Amphiphilic peptides form associates in water, such as micelles and vesicles, as a result of the self-assembly of single polymer chains [1]. Peptide-based associates have a potential use as drug carriers that enable the encapsulation or controlled release of drugs. For the innovation of pharmacological therapies, extensive efforts have been devoted to the creation of peptide-based drug carriers using amphiphilic peptides with the rational permutation of hydrophobic and hydrophilic amino acids [2,3,4,5]. However, the synthesis of amphiphilic peptides is based on labor-intensive, stepwise condensation reactions of amino acids, which restricts industrial applications. The bottom-up examination of self-assembled peptides strongly requires a simple structural motif for the systematic investigation and tuning of properties because it includes comprehensive studies to evaluate the correlations between (i) the amino acid sequence, (iii) the quasi-stable native 3D structure, (iii) the thermally stable structure perturbed by external stimuli, and (iv) the properties of associates.

**Figure 1:**
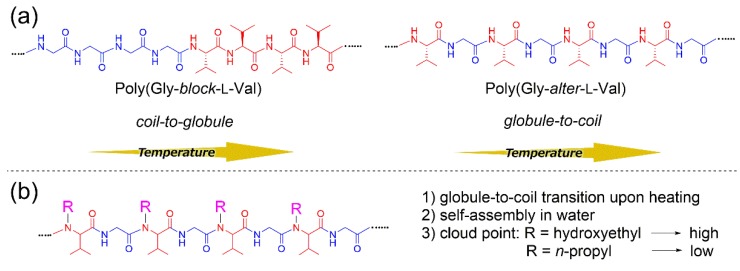
(**a**) Thermo-responsiveness of poly(Gly–*block*–L-Val) as a diblock copolypeptide and poly(Gly–*alter*–L-Val) as an alternating peptide (previous work) and (**b**) self-assembly and thermo-responsive properties of alternating peptides (current work).

Recently, we reported the impact of the amino acid sequence on peptide structure in aqueous solution and investigated the thermo-responsive behaviors of diblock, random, and alternating copolypeptides consisting of stoichiometric amounts of l-valine (l-Val) as a hydrophobic unit and glycine (Gly) as a hydrophilic unit (Figure 1a) [6]. The aqueous polymer solutions exhibited different thermal responsivity depending on the polymer sequence. It was found that the UV spectra of a diblock copolymer at various temperatures exhibited a cloud point, indicating that the polymer adopted coil-to-globule transition upon heating. The alternating peptide showed an upper critical solution temperature (UCST), which was found to be independent of the chirality of repeating units but influenced by the alternating binary pattern of hydrophobic and hydrophilic units. On the other hand, a random copolypeptide exhibited not only coil-to-globule transition at low temperatures but also globule-to-coil transition at high temperature owing to the presence of both segregated and alternating polymer sequences in the polymer.

In this work, we studied the self-assembly and thermo-responsive properties of newly synthesized poly(Gly–*alter*–Val) derivatives (Figure 1b). For the exploitation of the micellar properties of alternating peptides, we designed and synthesized *N*-substituted poly(Gly–*alter*–Val) derivatives and investigated the dependence of the hydrophilic–lipophilic balance of alternating peptides on the micellization behavior. It turned out that all the aqueous solutions of alternating peptides exhibited UCST behaviors, strongly indicating that the alternating binary pattern would contribute to the UCST behaviors. By the introduction of *n*-propyl groups as a hydrophobic functionality to the side chains, the polymers showed a cloud point in high-temperature regions. It was found that the cloud points of alternating peptides shifted to a higher temperature as the side chains became more hydrophilic, which is clearly opposite to the trend of typical surfactants [7]. We discuss the unusual micellization behaviors of alternating peptides through the estimation of single polymer chain structures in water.

## 2. Results and Discussion

**Scheme 1:**
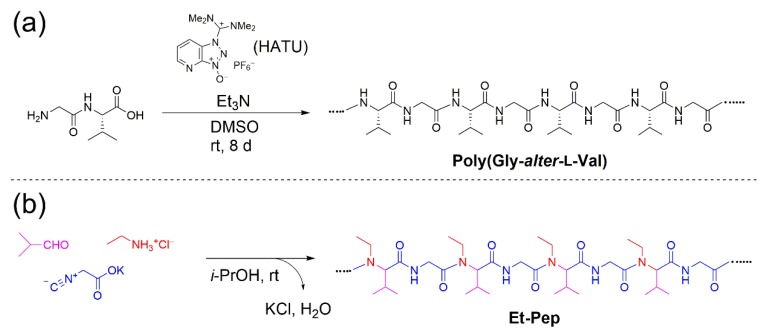
(**a**) Synthesis of poly(Gly–*alter*–L-Val) as alternating peptides via conventional polycondensation of a dipeptide monomer and (**b**) one-pot synthesis of Et-Pep via three-component polymerization.

We first prepared an alternating peptide consisting of Gly and l-Val via the conventional polycondensation of glycyl-l-valine as a dipeptide monomer and *O*-(7-azabenzotriazole-1-yl)-*N*,*N*,*N*′,*N*′-tetramethyluronium hexafluorophosphate (HATU) as a dehydrator (Scheme 1a). Treatment of the dipeptide with HATU in the presence of Et_3_N in DMSO for 8 d at room temperature afforded poly(Gly–*alter*–l-Val) as a white solid. We next synthesized *N*-ethylated poly(Gly–*alter*–Val), Et-Pep according to our recently developed technique for the one-pot synthesis of alternating peptides, with a slight modification [8]. Since our reported procedure is based on the use of alkylammonium trifluoromethanesulfonate, the work-up absolutely requires water treatment to remove amphiphilic potassium trifluoromethanesulfonate from the products. Assuming that Et-Pep would be highly soluble in water, making the extraction of products difficult, we exploited ethylammonium hydrochloride in place of ethylammonium trifluoromethanesulfonate as the reaction component of the three-component polymerization. A mixture of three components, i.e., isobutyraldehyde, ethylammonium hydrochloride, and potassium isocyanoacetate in *i*-PrOH was stirred for 8 d at room temperature and concentrated in vacuo. The resulting crude mixture was further stirred for 6 d at room temperature and afforded a highly viscous material. Tetrahydrofuran (THF) was added to the crude mixture to precipitate KCl, and the mixture was filtered and concentrated in vacuo to give *N*-ethylated poly (Gly–*alter*–Val), Et-Pep, as an amorphous compound, in the quantitative yield (Scheme 1b). The structure of Et-Pep was found to be an exactly alternating peptide structure, which was confirmed by the ^1^H NMR, ^13^C NMR, and IR spectra (Figures S4, S5 and S8) according to the literature [8]. The results suggest that the use of alkylammonium chloride makes the work-up process easy, without serious influence on the polymerization results.

**Figure 2:**
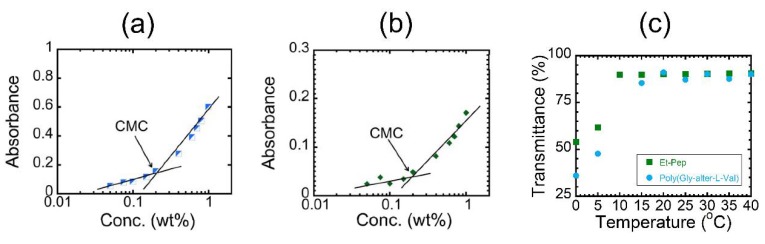
The absorbance of (**a**) poly(Gly-*alter*-L-Val) and (**b**) Et-Pep as a function of concentration (wt %) at 25 °C and (**c**) the temperature-dependence of transmittance of poly(Gly-*alter*-L-Val) (blue circle) and Et-Pep (green square) at 450 nm (1.5 wt %).

We measured the UV-vis spectra of these polymers at various concentrations to know the critical micelle concentration (CMC) (Figures S24 and S25), i.e., the onset concentration to form micelles according to the literature [9,10,11,12,13,14,15]. The absorbance was plotted against the logarithm of polymer concentration (Figure 2a,b). Each figure clearly includes an intersection point as the CMC of the polymers in water, supporting the formation of micelles. The CMCs were found to be approximately 0.2 wt % for the two polymers, indicating that the *N*-ethyl substitutions on the poly(Gly–*alter*–Val) skeleton hardly affected the CMC value. We next measured the UV-vis spectra of the aqueous solutions at various temperatures [10] and plotted the dependence of transmittance at 450 nm on temperature (Figure 2 and Figure S26). The plots indicated that both solutions exhibited a UCST-like transition. In the plots, the onset temperature to become translucent upon cooling is defined as a cloud point. The cloud points of both alternating peptides appear at below room temperature. The results confirmed that the regularly arranged binary pattern of hydrophobic and hydrophilic units in the alternating peptide would contribute to such UCST behaviors. Interestingly, the cloud point of poly(Gly–*alter*–L-Val) appeared at a higher temperature than that of Et-Pep, despite Et-Pep having hydrophobic alkyl side chains.

**Figure 3:**
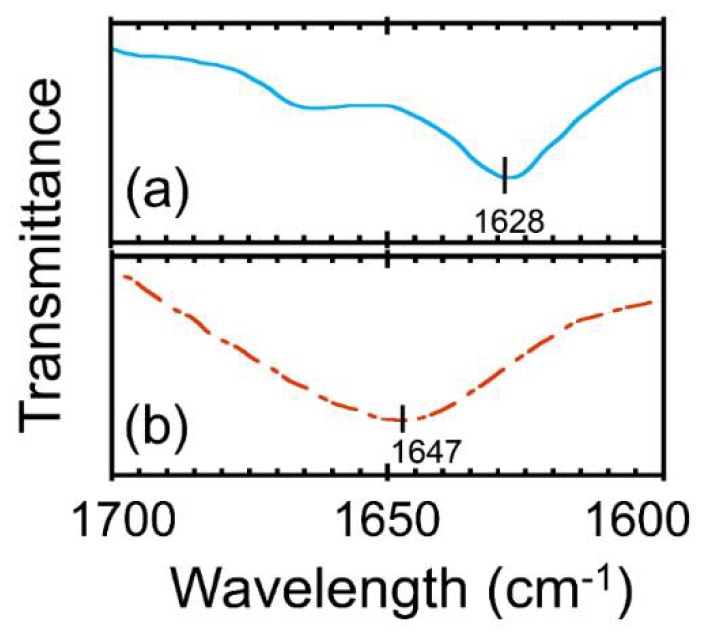
Partial IR spectra of (**a**) poly(Gly–*alter*–L-Val) and (**b**) Et-Pep (attenuated total reflection, ATR).

To know the correlations between the structures and the cloud points described above, we compared the IR spectra of the polymers (Figures S3 and S8). The partial IR spectra in a range of 1700–1600 cm^−1^ are shown in Figure 3. In the IR spectrum of poly(Gly–*alter*–L-Val), the amide carbonyl signal appears at 1628 cm^−1^, which is in a good agreement with the amide wavelength originating from a β-sheet structure [16,17], indicating the formation of a well-packed β-sheet structure in poly(Gly–*alter*–L-Val). On the other hand, the signal of Et-Pep is at 1647 cm^−1^, which is attributed to an α-helix or a random-coil structure. Since the regulation of chiral stereo-centers on Et-Pep was not considered during the polymerization reaction, the IR absorption wavelength of the amide carbonyl group suggests that Et-Pep adopts a flexible random-coil structure. The ethyl substitutions on the amides of Et-Pep may disturb hydrogen bond formation in the β-sheet structure. In addition, the main chain structure of Et-Pep includes *N*,*N*-disubstituted amides. It has been reported that the *N*,*N*-disubstitution of amides remarkably decreases the *trans*–*cis* rotation energy [18,19]. Therefore, the low rotation energy of *N*,*N*-disubstituted amides might also facilitate the formation of the random-coil structure. The results imply that the rigid β-sheet formation in the polymer on the interface upon cooling could aid the association of peptide amphiphiles [20].

**Scheme 2:**
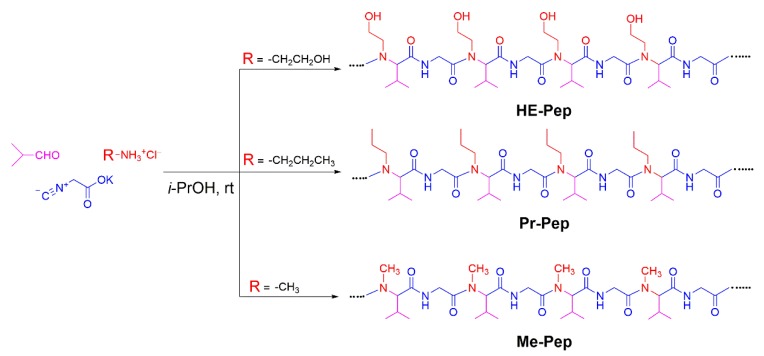
Synthesis of alternating peptide-based amphiphiles with different side chains.

For the creation of alternating peptides with a lower cloud point than that of Et-Pep, we decided to synthesize HE-Pep, with hydroxyl groups in the side chains as a polar functionality (Scheme 2). We also prepared Pr-Pep and Me-Pep as control samples. According to the synthetic procedure shown in Scheme 1, the treatment of isobutyraldehyde, potassium isocyanide, and the corresponding alkylammonium hydrochlorides afforded HE-Pep, Pr-Pep, and Me-Pep as alternating peptides in quantitative yields (Figures S9, S14 and S19). In the IR spectra of three polymers (Figures S13, S18 and S23), the amide carbonyl signals appeared at around 1647 cm^−1^ without a shoulder at 1628 cm^−1^, indicating that the polymers adopted a random-coil structure as a native structure.

**Figure 4:**
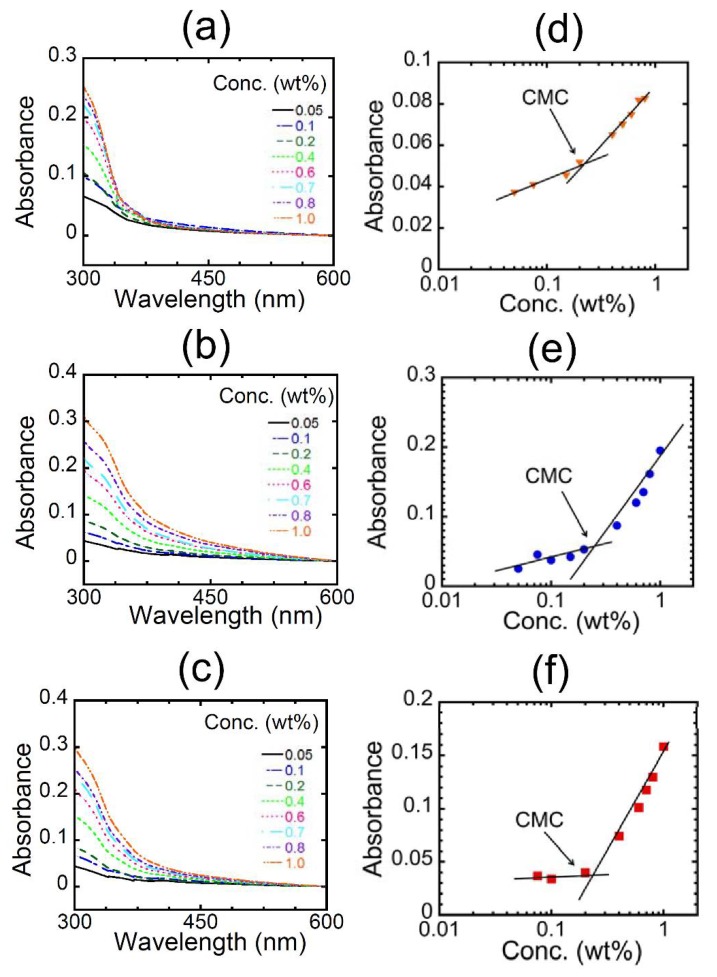
UV-vis spectra of (**a**) HE-Pep, (**b**) Pr-Pep, and (**c**) Me-Pep at various concentrations and the absorbance of (**d**) HE-Pep (orange triangle), (**e**) Pr-Pep (blue circle), and (**f**) Me-Pep (red square) at 340 nm as a function of concentration (wt %) at 25 °C.

With three polymers in hand, we estimated the CMC values by the UV-vis spectral measurements of the polymers in water (Figure 4). The absorbance was plotted against the logarithm of polymer concentration. Each figure clearly includes an intersection point as the CMC of the polymers in water, indicating the micellization of the polymers above the CMC. The CMCs were found to be approximately 0.2 wt % for all polymers, suggesting the independence of the structures of *N*-substituents on the CMC value.

**Figure 5:**
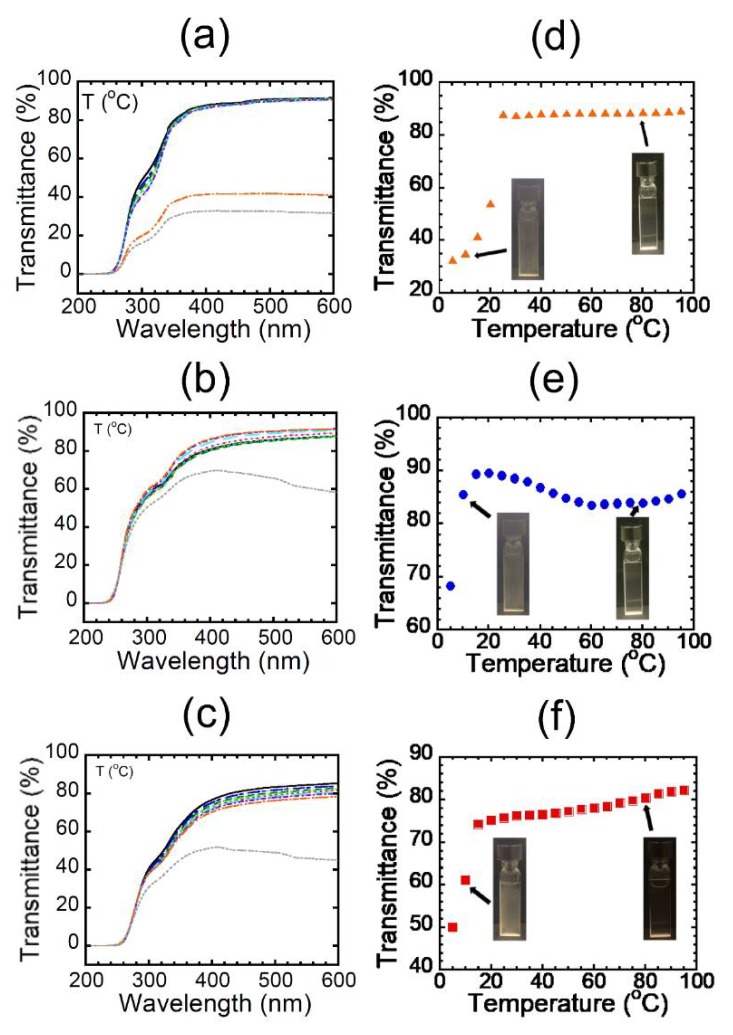
UV-vis spectra of (**a**) HE-Pep, (**b**) Pr-Pep, and (**c**) Me-Pep at various temperatures and the temperature dependence of transmittance of (**d**) HE-Pep (orange triangle), (**e**) Pr-Pep (blue circle), and (**f**) Me-Pep (red square) at 450 nm (1.5 wt %).

We next measured the UV–vis spectra of the aqueous solutions at various temperatures and plotted the dependence of transmittance at 450 nm on temperature (Figure 5). All the aqueous solutions exhibited UCST behaviors, strongly providing evidence that the alternating binary pattern would induce UCST behaviors. Curiously, it was found that the aqueous solutions of HE-Pep exhibited a clearly higher cloud point than that of Et-Pep (Figure 5d), despite HE-Pep comprising hydroxyl groups as a hydrophilic functionality. Furthermore, the cloud point of Pr-Pep appeared at a lower temperature than that of Et-Pep (Figure 5e), in spite of the longer hydrophobic side chains of Pr-Pep compared to Et-Pep. The results suggest some special polymer effects of the alternating peptide skeleton that reverses the trend of UCST-based cloud points [21,22]. At this moment, we noticed that the tendency observed in Figure 2c was not dependent only on the difference of secondary structures of the alternating peptides. Aside from the cloud point, the transmittance of Pr-Pep slightly decreased at the higher temperatures compared with that at around 20 °C, implying that the thermal aggregates of hydrophobic side chains would afford an additional cloud point upon heating [23]. The cloud point of Me-Pep appeared at a slightly higher temperature than that of Et-Pep (Figure 5f).

For the elucidation of such unusual tendency of cloud points, we next estimated the difference of hydrodynamic radius (*R*_h_) between HE-Pep and Pr-Pep [24]. With the use of an Einstein−Stokes equation (*R*_h_ = *k*_B_T/6πη*D*, *k*_B_: Boltzmann constant, T: absolute temperature, η: viscosity, *D*: diffusion coefficient), we performed diffusion-ordered NMR spectroscopic (DOSY) measurements to evaluate the diffusion coefficients of each polymer at 25 °C in both D_2_O (Figures S2, S7, S12, S17 and S22) and CF_3_COOD (trifluoroacetic acid, TFA-*d*, Figures S1, S6, S11, S16 and S21). It has been reported that typical polypeptides adopt a random-coil structure in TFA. The *D* values in D_2_O were estimated by using 0.15 wt % polymer solutions below the CMCs, considering the estimation of size for single polymer chains. The *D* values of a chosen chemical shift in the DOSY spectra were extracted by using the inversion of Laplace transform driven by the Dynamics center software (Bruker). By introducing the obtained *D* values in the Einstein–Stokes equation, the *R*_h_ values were estimated and are summarized in Table 1. The *R*_h_ value in D_2_O (*R*_water_) for HE-Pep was found to be of the same order to that in TFA-*d* (*R*_TFA_). On the other hand, the *R*_water_ of Pr-Pep was clearly lower than the *R*_TFA_ of Pr-Pep. The results indicate that Pr-Pep may adopt a globular structure in D_2_O, while HE-Pep could form a relatively extended structure such as a random-coil structure even in D_2_O.

Considering the solvent dependency of the *R*_h_ values, the trend of UCST-based cloud points of alternating peptides seems to be correlated to the quasi-stable single polymer structures in water. The structure of HE-Pep presents regularly aligned hydroxyl groups in the same direction and can be regarded as a Janus structure [25]. The hydrophobic alkyl chains may be exposed in an organic solvent, which could be accompanied by the association of hydroxyl group-containing side chains. In contrast, the hydroxyl group-containing side chains could be projected outward in water along with the association of hydrophobic alkyl chains. As a result, the Janus structure of HE-Pep would make the *R*_h_ values in an organic solvent and water similar. Since the peptide main chain of Pr-Pep as an organophobe would be efficiently covered by the hydrophobic side chains, Pr-Pep is expected to adopt an extended structure in an organic solvent. However, the hydrophilic main chain of Pr-Pep would be exposed in water along with the aggregation of hydrophobic side chains, which could be accompanied by the *trans*–*cis* isomerization of *N*,*N*-disubstituted amides because of the low rotation energy. The structural isomerization of Pr-Pep should be a plausible reason for the contraction of the polymer in water. In addition to the hydrophilic–lipophilic balance of alternating peptides, the morphology of quasi-stable single polymer chains in water would be related to the opposite trend of UCST-based cloud points. HE-Pep could adopt a relatively extended structure in water compared with the other *N*-substituted alternating peptides, which might facilitate the formation of second associates upon cooling, leading to the high cloud point of HE-Pep. Detailed studies of the morphology of single polymer chains will be performed as a part of our future work.

## 3. Materials and Methods

Glycyl–l-valine (Tokyo Chemical Industry Co., Ltd., Tokyo, Japan), *O*-(7-azabenzotriazole-1-yl)-*N*,*N*,*N*′,*N*′-tetramethyluronium hexafluorophosphate (HATU, Tokyo Chemical Industry Co., Ltd.), isobutyraldehyde (Tokyo Chemical Industry Co., Ltd., Tokyo, Japan), ethylammonium chloride (FUJIFILM Wako Pure Chemical Corporation, Osaka, Japan), *I*-propyl alcohol (Taiyo Chemicals, Co., Ltd., Wakayama, Japan), 2-aminoethanol (FUJIFILM Wako Pure Chemical Corporation, Osaka, Japan), propylammonium chloride (FUJIFILM Wako Pure Chemical Corporation, Osaka, Japan), methylammonium chloride (FUJIFILM Wako Pure Chemical Corporation, Osaka, Japan) were used as obtained. Potassium isocyanoacetate was synthesized according to the literature [26].

The ^1^H NMR (400 MHz) and ^13^C NMR (100 MHz) spectra were recorded on a Bruker Avance II 400 spectrometer (Bruker, Fällanden, Switzerland), using CDCl_3_ as a solvent, and calibrated using residual undeuterated solvent and tetramethylsilane as internal standards. Diffusion-ordered NMR spectroscopic analyses (DOSY) were carried out using the alternating peptides in CF_3_COOD (TFA-*d*) or in D_2_O at highly diluted concentrations to estimate the diffusion coefficients. Experiments were run without spinning to avoid convection. The standard Bruker pulse program ledbpgp2s was used, which employs simulated echo and longitudinal eddy delay with bipolar gradients and two spoil gradients. The diffusion coefficients of a selected narrow chemical shift in the spectra of the compounds were determined using the Dynamics Center software (ver. 2.4.8; Bruker, Fällanden, Switzerland). The calibration line in TFA-*d* for *M*_w_ prediction was obtained by the diffusion coefficients of angiotensin II (MW: 1064.18, log *D*: −9.38), insulin (MW: 5807.57, log *D*; −9.77) and cytochrome C (MW: 12384, log *D*: −9.87) as standards [8,27]. Fourier-transform infrared (FTIR) spectra of the samples in solid state were recorded via an attenuated total reflection (ATR) method using a PerkinElmer spectrum 100 spectrometer (PerkinElmer, Shelton, USA). UV-vis spectra were recorded on a JASCO V-550 spectrophotometer (JASCO Co. Ltd., Tokyo, Japan) using a 1.0 mm path quartz cell with a temperature controller (ETC-505T, JASCO Co. Ltd., Tokyo, Japan).

### 3.1. Synthesis of Poly(Gly–alter–l-Val)

Et_3_N (1.91 mL, 13.8 mmol) and HATU (4.92 g, 12.9 mmol) were added to a solution of glycyl–l-valine (1.5 g, 8.61 mmol) in DMSO (8.4 mL) at room temperature. After stirring for 8 d, MeOH (90 mL) was added to the mixture. The mixture was sonicated and filtered to collect white solids, which were further washed with MeOH and CHCl_3_ and dried in vacuo to give poly(Gly–*alter*–l-Val) (1.72 g) in quantitative yield: *M*_w_ 2,900 Da (estimated by NMR, Figure S1); IR (ATR) υ 3278 (NH), 2965, 1628 (C=O), 1534, 1498, 1457, 1432, 1411, 1323, 1272, 1222, 1180, 1109, 1041, 1026, 842, 706 cm^−1^ (Figure S3).

### 3.2. Synthesis of Et-Pep

Ethylammonium chloride (463 mg, 5.68 mmol) was added to *i*-PrOH (2.9 mL), followed by the addition of isobutylaldehyde (519 μL, 5.68 mmol) and potassium isocyanate (700 mg, 5.68 mmol). The reaction mixture was dissolved by sonication. The mixture was stirred for 8 d at room temperature and concentrated in vacuo. The resulting crude mixture was further stirred for 6 d at room temperature and diluted with THF to precipitate inorganic salts. The mixture was filtered and concentrated in vacuo to give the corresponding alternating peptide (Et-Pep, 1.12 g) in quantitative yield: *M*_w_ 1000 Da (estimated by NMR, Figure S6); ^13^C NMR (100 MHz, 298 K, CDCl_3_) δ 172–160 (C=O), 68.0 (CH), 43.4–43.3 (CH_2_), 34.8–30.4 (CH), 27.4–13.4 (CH_2_, CH_3_) ppm (Figure S5); IR (ATR) *υ*3281 (NH), 2968, 2875, 1647 (C=O), 1533, 1466, 1381, 1264, 1233, 1105, 930 cm^−1^ (Figure S8).

### 3.3. Synthesis of Pr-Pep

Propylammonium chloride (543 mg, 5.68 mmol) was added to *i*-PrOH (2.9 mL), followed by the addition of isobutylaldehyde (519 μL, 5.68 mmol) and potassium isocyanate (700 mg, 5.68 mmol). The reaction mixture was dissolved by sonication. The mixture was stirred for 8 d at room temperature and concentrated in vacuo. The resulting crude mixture was further stirred for 6 d at room temperature and diluted with THF to precipitate inorganic salts. The mixture was filtered and concentrated in vacuo to give the corresponding alternating peptide (Pr-Pep, 1.24g) in quantitative yield: *M*_w_ 800 Da (estimated by NMR, Figure S16); ^13^C NMR (100 MHz, 298 K, CDCl_3_) δ 174–160 (C=O), 68.2–67.3 (CH), 50.5–41.5 (CH_2_), 30.7–25.8 (CH), 22.9–11.6 (CH_2_, CH_3_) ppm (Figure S15); IR (ATR) *υ*3281 (NH), 2963, 2876, 1647 (C=O), 1533, 1466, 1382, 1229, 1105, 1028, 958 cm^−1^ (Figure S18).

### 3.4. Synthesis of HE-Pep

The compound 2-aminoethanol (0.69 mL, 11.5 μmol) was treated with 1.0 M HCl aq. (12 mL) at 0 °C, concentrated in vacuo, and dried under reduced pressure at 70 °C to give 2-aminoethanol hydrochloride. The obtained 2-aminoethanol hydrochloride (544 mg, 5.68 mmol) was added to *i-*PrOH (2.9 mL), followed by the addition of isobutylaldehyde (519 μL, 5.68 mmol) and potassium isocyanate (700 mg, 5.68 mmol). The reaction mixture was dissolved by sonication. The mixture was stirred for 8 d at room temperature and concentrated in vacuo. The resulting crude mixture was further stirred for 6 d at room temperature and diluted with THF to precipitate inorganic salts. The mixture was filtered and concentrated in vacuo to give the corresponding alternating peptide (HE-Pep, 1.25g) in a quantitative yield: *M*_w_ 1,100 Da (estimated by NMR, Figure S11); ^13^C NMR (100 MHz, 298 K, CDCl_3_) δ 174–169 (C=O), 68.6–60.1 (CH), 47.7–42.2 (CH_2_), 30.5–29.2 (CH), 25.6–16.6 (CH_2_, CH_3_) ppm (Figure S10); IR (ATR) *υ* 3287 (NH), 2962, 2873, 1647 (C=O), 1539, 1466, 1387, 1188, 1039, 1029, 959 cm^−1^ (Figure S13).

### 3.5. Synthesis of Me-Pep

Methylammonium chloride (384 mg, 5.68 mmol) was added to –*i-*PrOH (2.9 mL), followed by the addition of isobutylaldehyde (519 μL, 5.68 mmol) and potassium isocyanate (700 mg, 5.68 mmol). The reaction mixture was dissolved by sonication. The mixture was stirred for 8 d at room temperature and concentrated in vacuo. The resulting crude mixture was further stirred for 6 d at room temperature and diluted with THF to precipitate inorganic salts. The mixture was filtered and concentrated in vacuo to give the corresponding alternating peptide (Me-Pep, 1.05 g) in a quantitative yield: *M*_w_ 1000 Da (estimated by NMR, Figure S21); ^13^C NMR (100 MHz, 298 K, CDCl_3_) δ 174–162 (C=O), 75.2–68.1 (CH), 45.1–41.8 (CH_2_), 33.5–31.7 (CH), 22.6–16.8 (CH_3_) ppm (Figure S20); IR (ATR) *υ*3285 (NH), 2963, 1645 (C=O), 1531, 1468, 1410, 1386, 1230, 1164, 1104, 1027 cm^−1^ (Figure S23).

### 3.6. Determination of Critical Micelle Concentration (CMC)

Stock solutions of the polymer samples were prepared by dissolution in H_2_O. The aqueous solutions at various concentrations were prepared using the stock solution by proper dilution. The UV-vis spectra of the sample solutions were recorded on a JASCO V-550 spectrophotometer (JASCO Co. Ltd., Tokyo, Japan) using a 1.0 mm path quartz cell at 25 °C. The obtained absorbance values at 340–350 nm were standardized by the concentration and were plotted as a function of the concentration. From the intersection point in the plots, we determined the CMC values of the polymers in water.

### 3.7. Determination of Cloud Point 

The cloud points of the polymers were determined by the UV-vis spectra of 1.5 wt % polymer aqueous solutions at various temperatures (5–95 °C). The obtained transmittance values were plotted as the function of temperature. From the plots, we determined the cloud points of the polymers as the onset temperature to become translucent upon cooling.

### 3.8. Estimation of the Hydrodynamic Radius

We measured DOSY spectra using solutions at various concentrations in aqueous (D_2_O) and organic solvents (TFA-*d*) to estimate the diffusion coefficients (*D*). For example, the DOSY spectrum was obtained using HE-Pep (0.9 mg) in D_2_O (0.6 mL) to estimate the *D* value for single polymer chains at 298 K (Figure S12). The experiment was run without spinning to avoid convection. The standard Bruker pulse program ledbpgp2s was used, which employs simulated echo and longitudinal eddy delay with bipolar gradients and two spoil gradients. The diffusion coefficients of a selected narrow chemical shift in the spectra of the compounds were extracted by using the inversion of Laplace transform driven by the Dynamics Center software (ver. 2.4.8; Bruker, Fällanden, Switzerland). By introducing the obtained *D* values in the Einstein–Stokes equation (*R*_h_ = *k*_B_T/6πη*D*, *k*_B_: Boltzmann constant, T: absolute temperature, η: viscosity, *D*: diffusion coefficient), we estimated the *R*_h_ values of the polymers to be 0.581 ± 0.014 nm (Table 1). For the other polymers, we estimated the *R*_h_ values in a similar manner (Table 1 and Table S1).

## 4. Conclusions

In conclusion, we designed and investigated the micellization behaviors of alternating peptides in water. The effects of substituents on the amide nitrogen atoms were systematically investigated using the reliable polymer structure consisting of exactly alternating peptides. The alternating peptides were successfully prepared via three-component polymerization exploiting Ugi’s four-component polymerization as the elemental polymerization reaction. The micellization behaviors of alternating peptides were evaluated by analyzing their UV-vis spectra at various concentrations and temperatures and DOSY measurements in D_2_O and TFA-*d*. All the aqueous solutions of the polymers exhibited UCST behaviors. It turned out that the cloud points of alternating peptides shifted to higher temperatures as the side chains became more hydrophilic, which is clearly opposite to the trend of typical surfactants. The comparison of *R*_h_ values in D_2_O and TFA-*d* indicated that this opposite trend of cloud points is closely related to the regular alignment of *N*-substituents of alternating peptides. The present study provides new insights not only for the rational control of amphiphilicity of peptides but also for the control of their micellar properties. Due to the biodegradable nature [28] and controlled micellar properties, these peptides might have applications as drug carriers in the field of pharmaceutical engineering.

## Figures and Tables

**Table 1:** CMC, cloud point, and hydrodynamic radius (*R*_h_) of HE-Pep and Pr-Pep *^a^*.

Alternating Peptide	CMC (wt %)	Cloud Point (^°^C)	*R*_h_ in D_2_O (*R*_water_, nm)	*R*_h_ in TFA-*d* (*R*_TFA_, nm)	*R*_water_/*R*_TFA_
**HE-Pep**	0.2	25	0.581 ± 0.014	0.576 ± 0.004	1.01
**Pr-Pep**	0.2	15	0.442 ± 0.011	0.507 ± 0.005	0.87

*^a^* Standard deviations are from three samples. TFA: trifluoroacetic acid.

## Supplementary Materials

Supplementary materials can be found at https://www.mdpi.com/1422-0067/20/18/4604/s1.

## Abbreviations

UCST	Upper critical solution temperature
HATU	*O*-(7-Azabenzotriazole-1-yl)-*N*,*N*,*N*′,*N*′-tetramethyluronium hexafluorophosphate
CMC	Critical micelle concentration
DOSY	Diffusion-ordered NMR spectroscopy
TFA	Trifluoroacetic acid
D	Diffusion coefficient
*R*_h_	Hydrodynamic radius

## References

[1] Cavalli S., Albericio F., Kros A. (2010). Amphiphilic peptides and their cross-disciplinary role as building blocks for nanoscience. Chem. Soc. Rev..

[2] Hamley I.W. (2011). Self-assembly of amphiphilic peptides. Soft Matter.

[3] Dexter A.F., Middelberg A.P.J. (2008). Peptides as functional surfactants. Ind. Eng. Chem. Res.

[4] Dehsorkhi A., Castelletto V., Hamley I.W. (2014). Self-assembling amphiphilic peptides. J. Pept. Sci..

[5] Qin F., Chen Y., Tang C., Zhao X. (2018). Amphiphilic peptides as novel nanomaterials: Design, self-assembly and application. Int. J. Nanomed..

[6] Ihsan A.B., Koyama Y. (2019). Impact of polypeptide sequence on thermal properties for diblock, random, and alternating copolymers containing a stoichiometric mixture of glycine and valine. Polymer.

[7] Chanachichalermwong W., Charoensaeng A., Suriyapraphadilok U. (2019). Krafft point prediction of anionic surfactants using group contribution method: First-order and higher-order groups. J. Surfact. Deterg..

[8] Koyama Y., Gudeangadi P.G. (2017). One-pot synthesis of alternating peptides exploiting a new polymerization technique based on Ugi’s 4CC reaction. Chem. Commun..

[9] Cai L., Gochin M., Liu K. (2011). A facile surfactant critical micelle concentration determination. Chem. Commun..

[10] Nesměrák K., Němcová I. (2006). Determination of critical micelle concentration by electrochemical means. Anal. Lett..

[11] Mukerjee P., U.S. Department of Commerce (1971). Critical micelle concentrations of aqueous surfactant systems. National Standard Reference Data System, National Bureau of Standards.

[12] Li N., Luo H., Liu S. (2004). A new method for the determination of the critical micelle concentration of Triton X-100 in the absence and presence of β-cyclodextrn by resonance Rayleigh scattering technology. Spectrochim. Acta Part A Mol. Biomol. Spectrosc..

[13] Chan C.Y., Wang S.J., Liu I.J., Chiu Y.C. (1987). A simple method for determining the critical micellar concentration of a surfactant. J. Chin. Chem. Soc..

[14] Mandavi R., Sar S.K., Ranthore N. (2008). Critical micelle concentration of surfactant, mixed-surfactant and polymer by different method at room temperature and its importance. Orient. J. Chem..

[15] Ghosh S., Krishnan A., Das P.K., Ramakrishnan S. (2003). Determination of critical micelle concentration by hyper-Rayleigh scattering. J. Am. Chem. Soc..

[16] Oh H.J., Joo M.K., Sohn Y.S., Jeong B. (2008). Secondary structure effect of polypeptide on reverse thermal gelation and degradation of L/DL-poly(alanine)-poloxamer-L/DL-poly(alanine) copolymers. Macromolecules.

[17] Cheng Y., He C., Xiao C., Ding J., Zhuang X., Huang Y., Chen X. (2012). Decisive role of hydrophobic side groups of polypeptides in thermosensitive gelation. Biomacromolecules.

[18] Stewart W.E., Siddall T.H. (1970). Nuclear magnetic resonance studies of amides. Chem. Rev..

[19] Aya T., Isao A., Hiroyuki K. (2000). Aromatic architecture based on *cis* conformational preference of *N*-methylated amides. Yuki Gosei Kagaku Kyokaishi.

[20] Rapaport H., Kjaer K., Jensen T.R., Leiserowitz L., Tirrell D.A. (2000). Two-dimensional order in β-sheet peptide monolayers. J. Am. Chem. Soc..

[21] Kaewsaiha P., Matsumoto K., Matsuoka H. (2005). Non-surface activity and micellization of ionic amphiphilic diblock copolymers in water. Hydrophobic chain length dependence and salt effect on surface activity and the critical micelle concentration. Langmuir.

[22] Matsuoka H., Chen H., Matsumoto K. (2012). Molecular weight dependence of non-surface activity for ionic amphiphilic diblock copolymers. Soft Matter.

[23] Koyama Y., Ihsan A.B., Gudeangadi P.G. (2018). Synthetic approach of thermally tunable nature-mimetic polypeptides from *N*-protected alternating peptoids. Macromol. Chem. Phys..

[24] Ihsan A.B., Koyama Y., Taira T., Imura T. (2018). Thermo-responsive structure and surface activity of kinetically stabilized micelle composed of fluorinated alternating peptides in organic solvent. ChemistrySelect.

[25] Walther A., Müller A.H.E. (2013). Janus particles: Synthesis, self-assembly, physical properties, and applications. Chem. Rev..

[26] Bonne D., Dekhane M., Zhu J. (2004). Ammonium chloride promoted Ugi four-component, five-center reaction of α-substituted α-isocyano acetic acid: A strong solvent effect. Org. Lett..

[27] Li W., Chung H., Daeffler C., Johnson J.A., Grubbs R.H. (2012). Application of ^1^H DOSY for facile measurements of polymer molecular weights. Macromolecules.

[28] Tsuchiya K., Ifuku N., Koyama Y., Numata K. (2019). Development of regenerated silk films coated with fluorinated polypeptides to achieve high water repellency and biodegradability in seawater. Polym. Degrad. Stab..

