# Supplementary material for: Effects of the Hydrophilic–Lipophilic Balance of Alternating Peptides on Self-Assembly and Thermo-Responsive Behaviors
Source: Int J Mol Sci. 2019 Sep 17;20(18):4604. doi: 10.3390/ijms20184604 (PMC6770757; doi:10.3390/ijms20184604)
Supplement: Supplementary file 1 [file ijms-20-04604-s001.pdf]

Supplementary Materials

Effects of Hydrophilic-Lipophilic Balance of Alternating Peptides on Self-Assembly and Thermo-Responsive Behaviors

Abu Bin Ihsan, Mahmuda Nargis, Yasuhito Koyama*

Department of Pharmaceutical Engineering, Faculty of Engineering
Toyama Prefectural University, 5180 Kurokawa, Imizu, Toyama 939-0398, Japan
*E-mail: ykoyama@pu-toyama.ac.jp

Contents

DOSY and IR Spectra of Poly(Gly-*alter*-L-Val) 2

^{1}H NMR, ^{13}C NMR, DOSY, and IR Spectra of Et-Pep 5

^{1}H NMR, ^{13}C NMR, DOSY, and IR Spectra of HE-Pep 9

^{1}H NMR, ^{13}C NMR, DOSY, and IR Spectra of Pr-Pep 13

^{1}H NMR, ^{13}C NMR, DOSY, and IR Spectra of Me-Pep 17

UV–vis Spectra 21

Table for CMC, cloud point, and hydrodynamic radius 22

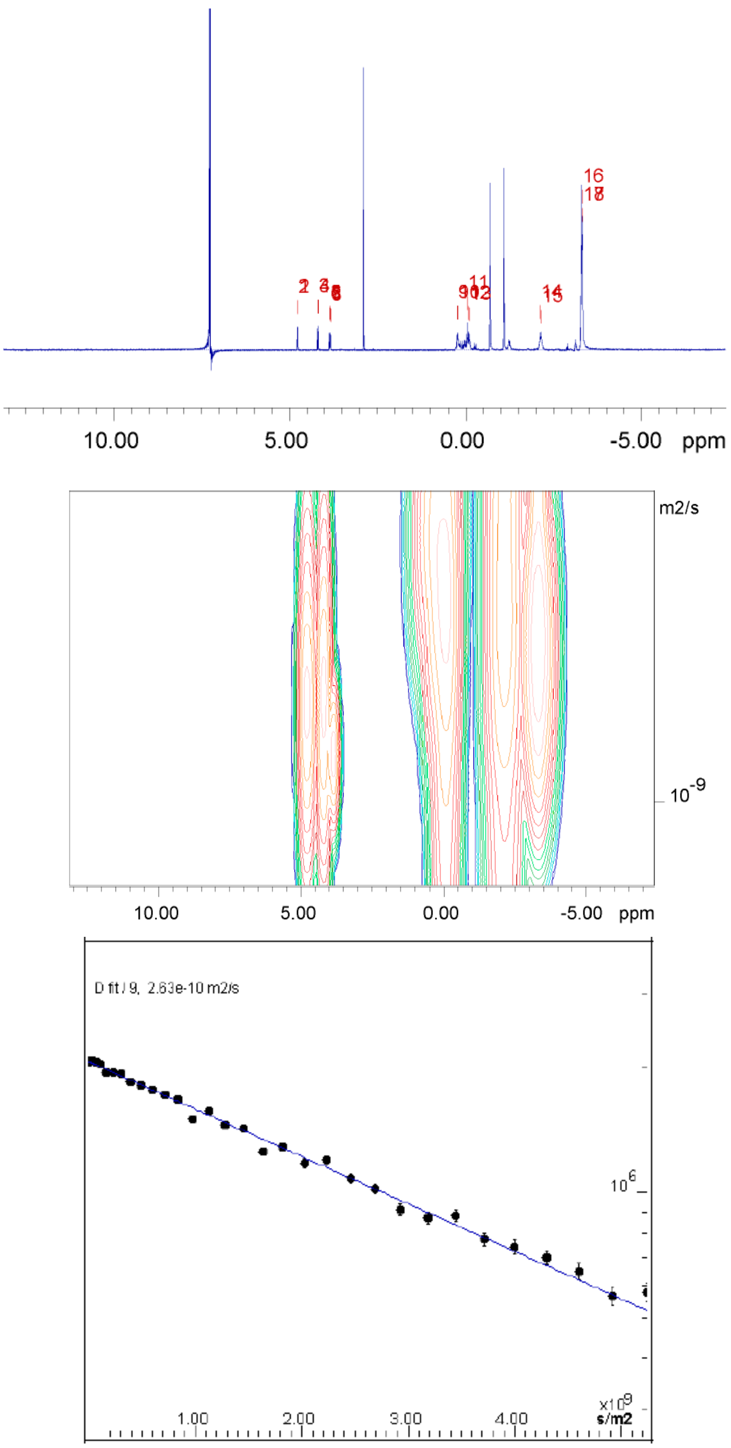

Figure S1: DOSY correlations of **Poly(Gly-*alter*-L-Val)** (400 MHz, TFA-*d*, 298 K) and the attenuation curve.

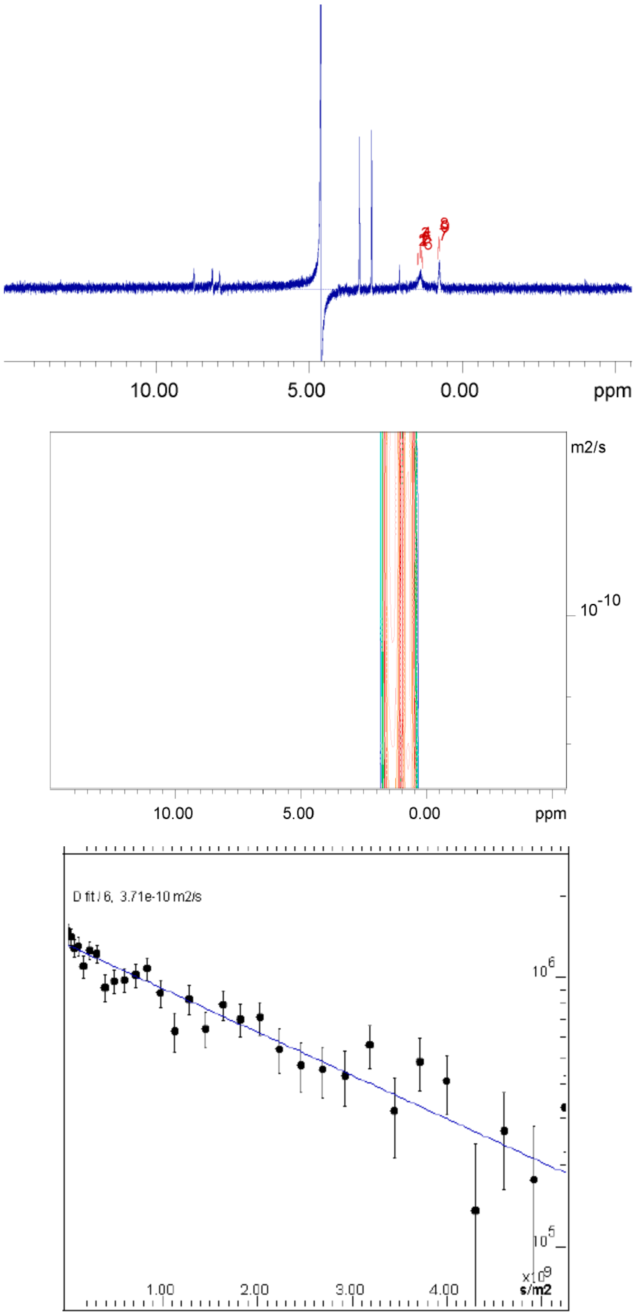

Figure S2: DOSY correlations of **Poly(Gly-*alter*-L-Val)** (400 MHz, D_2O, 298 K) and the attenuation curve.

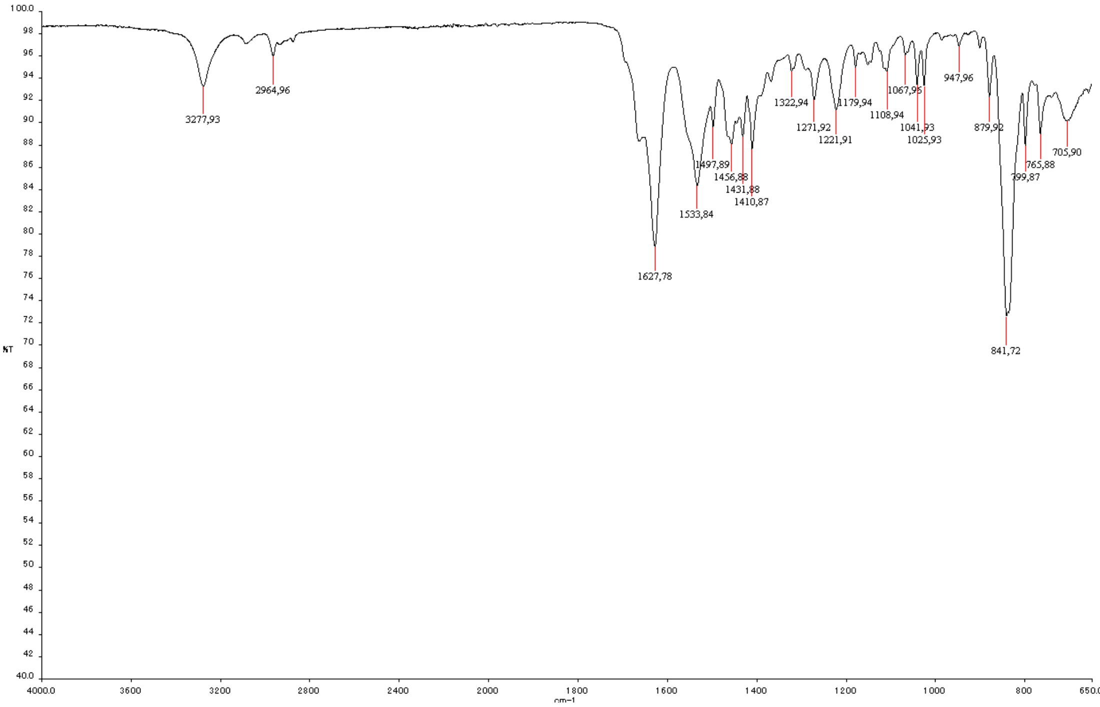

Figure S3: IR spectrum of **Poly(Gly-*alter*-L-Val)** (ATR).

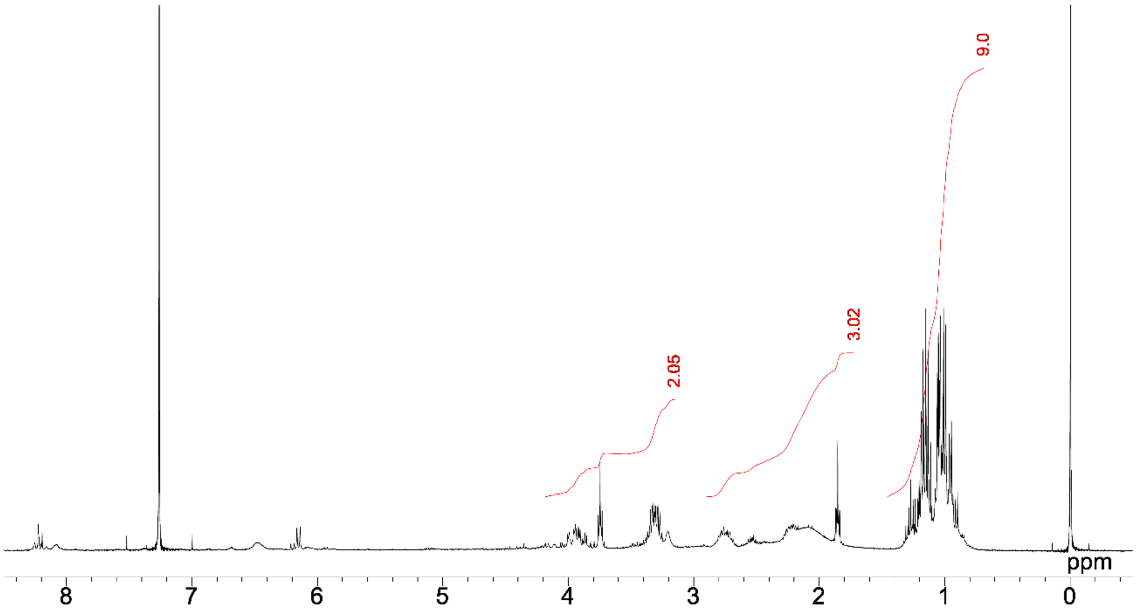

Figure S4: ^{1}H NMR spectrum of **Et-Pep** (400 MHz, $CDCl_3$, 298 K).

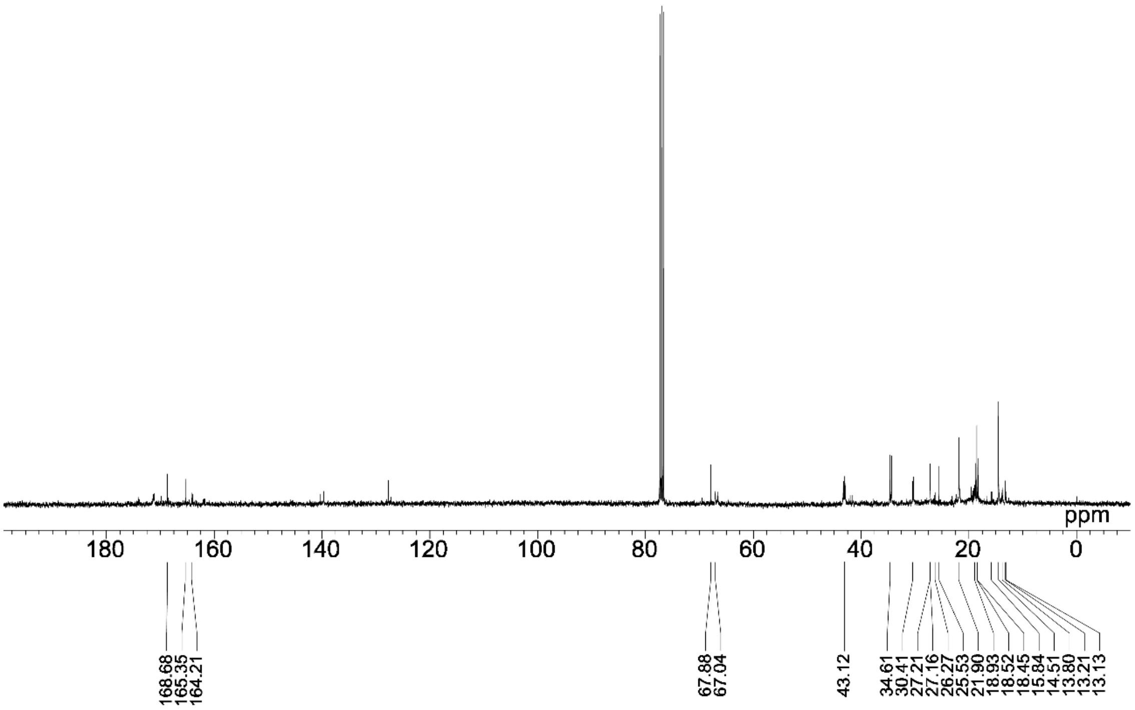

Figure S5: ^{13}C NMR spectrum of **Et-Pep** (100 MHz, $CDCl_3$, 298 K).

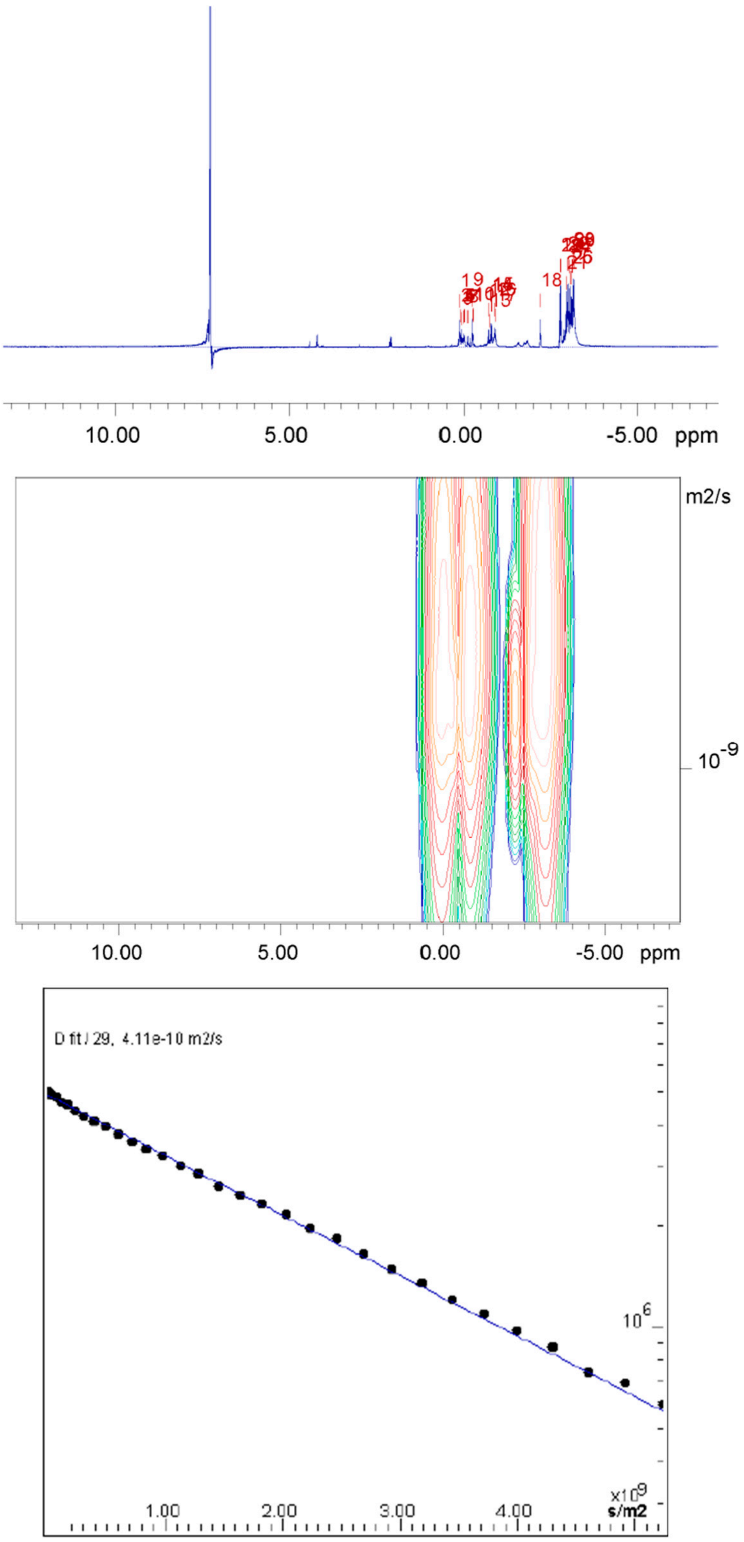

Figure S6: DOSY correlations of **Et-Pep** (400 MHz, TFA-*d*, 298 K) and the attenuation curve.

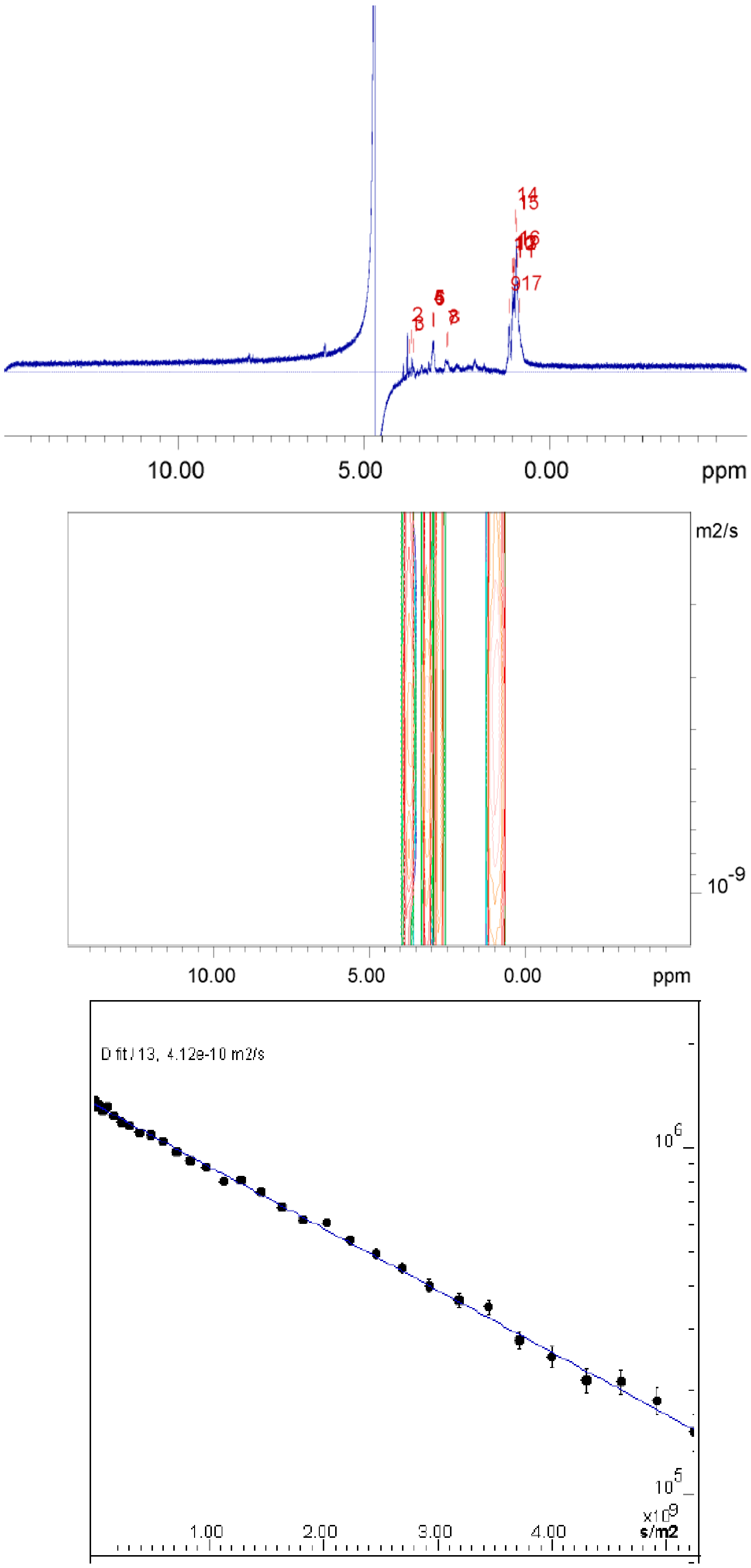

Figure S7: DOSY correlations of **Et-Pep** (400 MHz, D_2O, 298 K) and the attenuation curve.

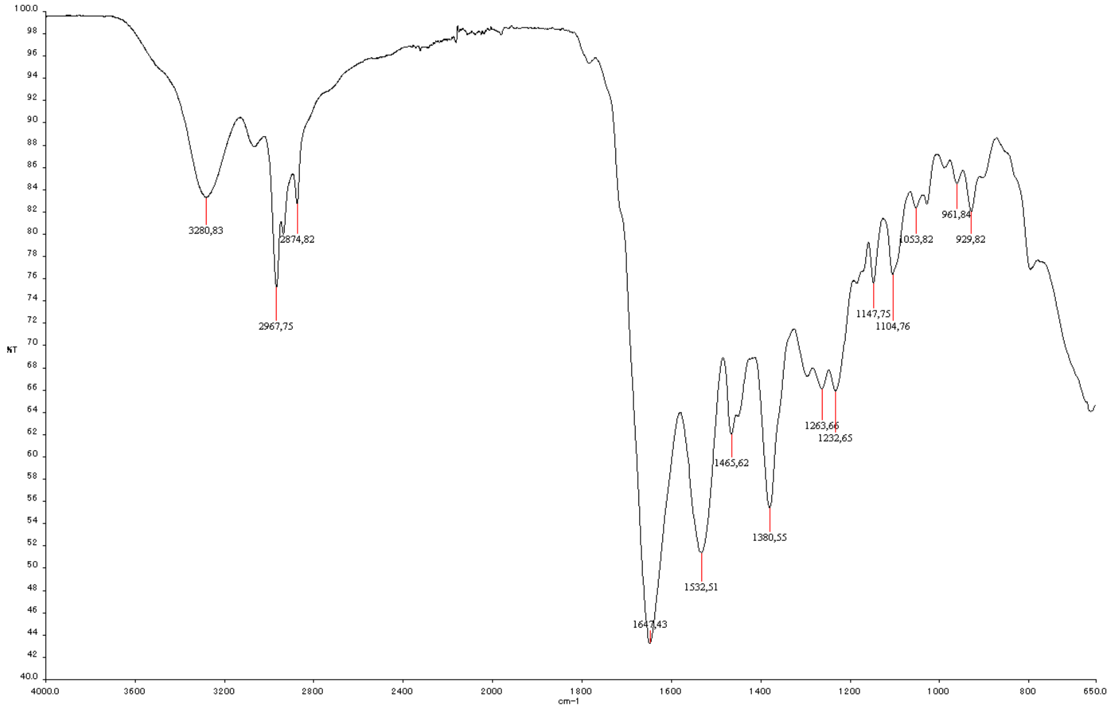

Figure S8: IR spectrum of **Et-Pep** (ATR).

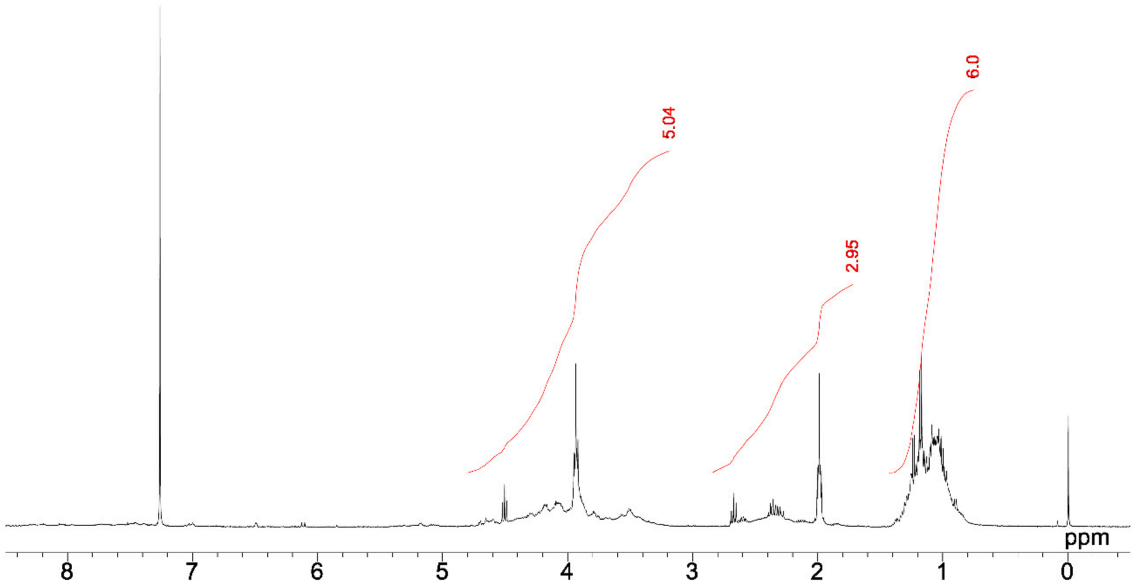

Figure S9: ^{1}H NMR spectrum of **HE-Pep** (400 MHz, $CDCl_3$, 298 K).

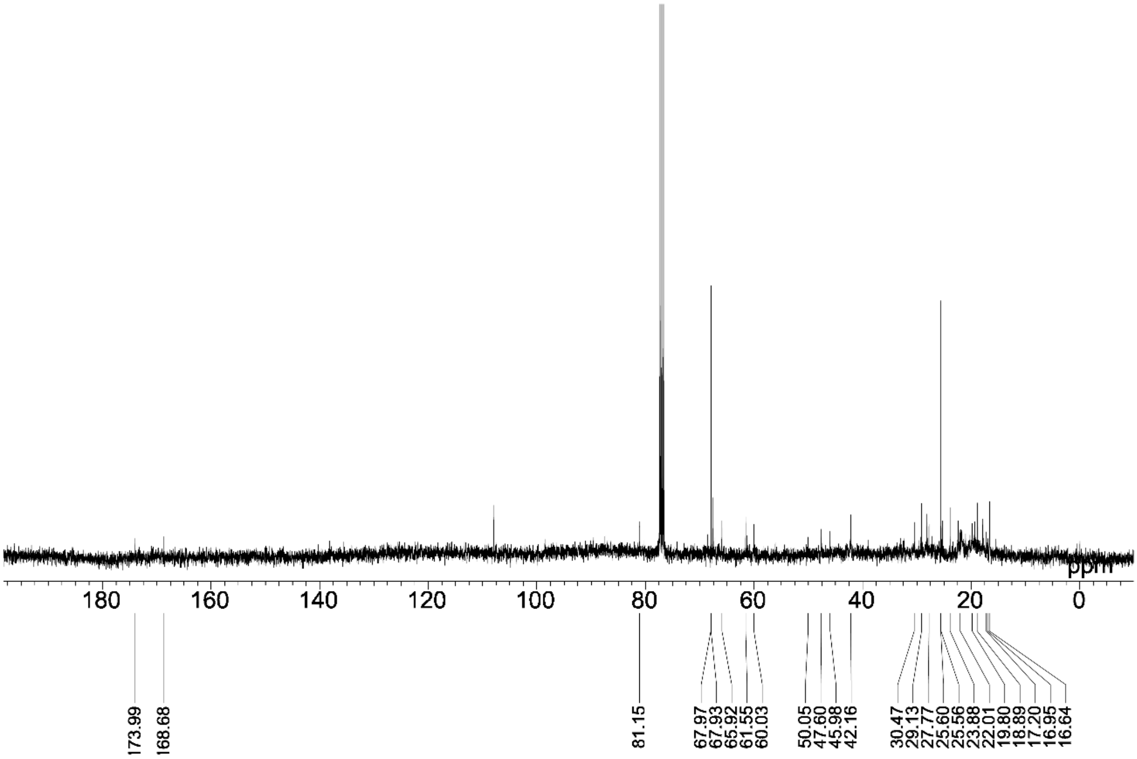

Figure S10: ^{13}C NMR spectrum of **HE-Pep** (100 MHz, $CDCl_3$, 298 K).

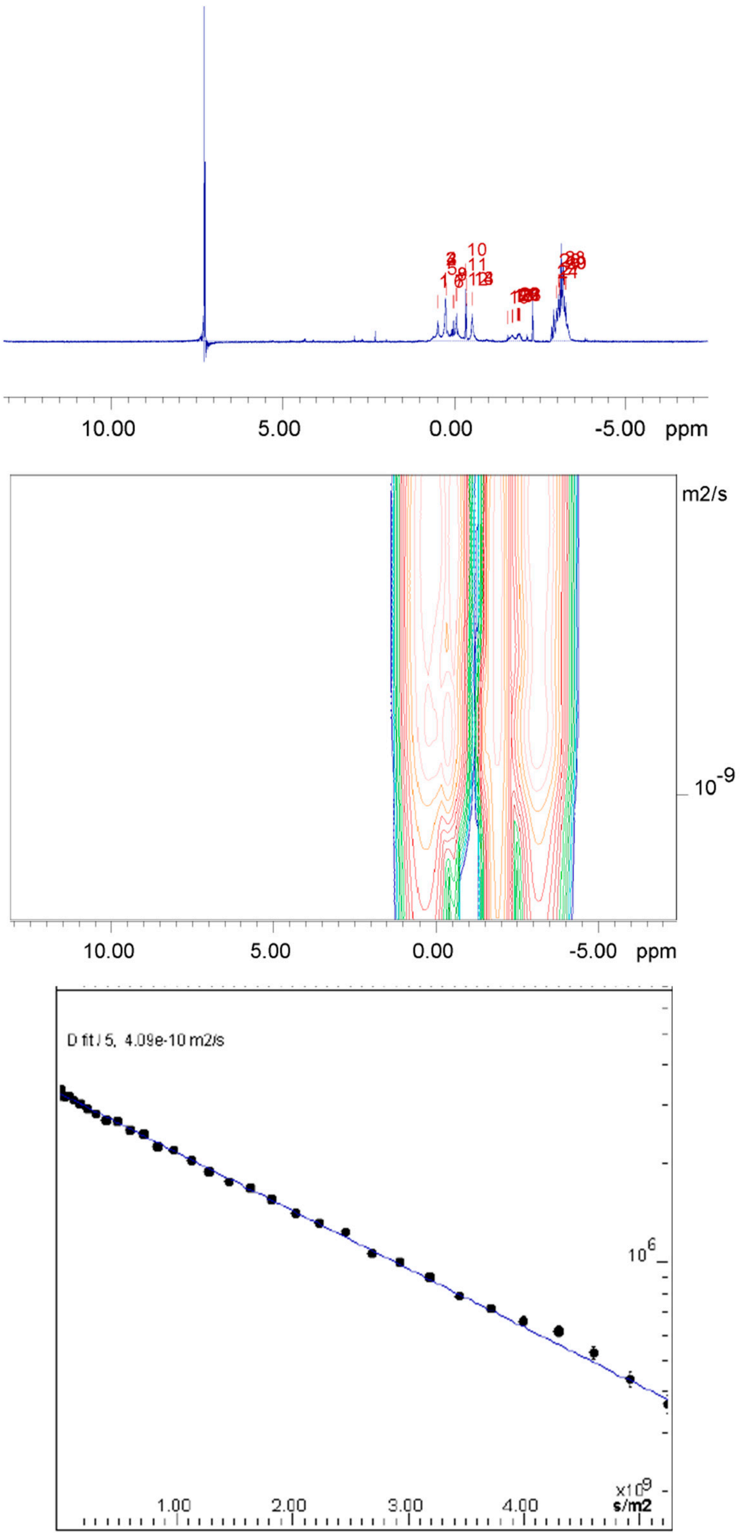

Figure S11: DOSY correlations of **HE-Pep** (400 MHz, TFA-*d*, 298 K) and the attenuation curve.

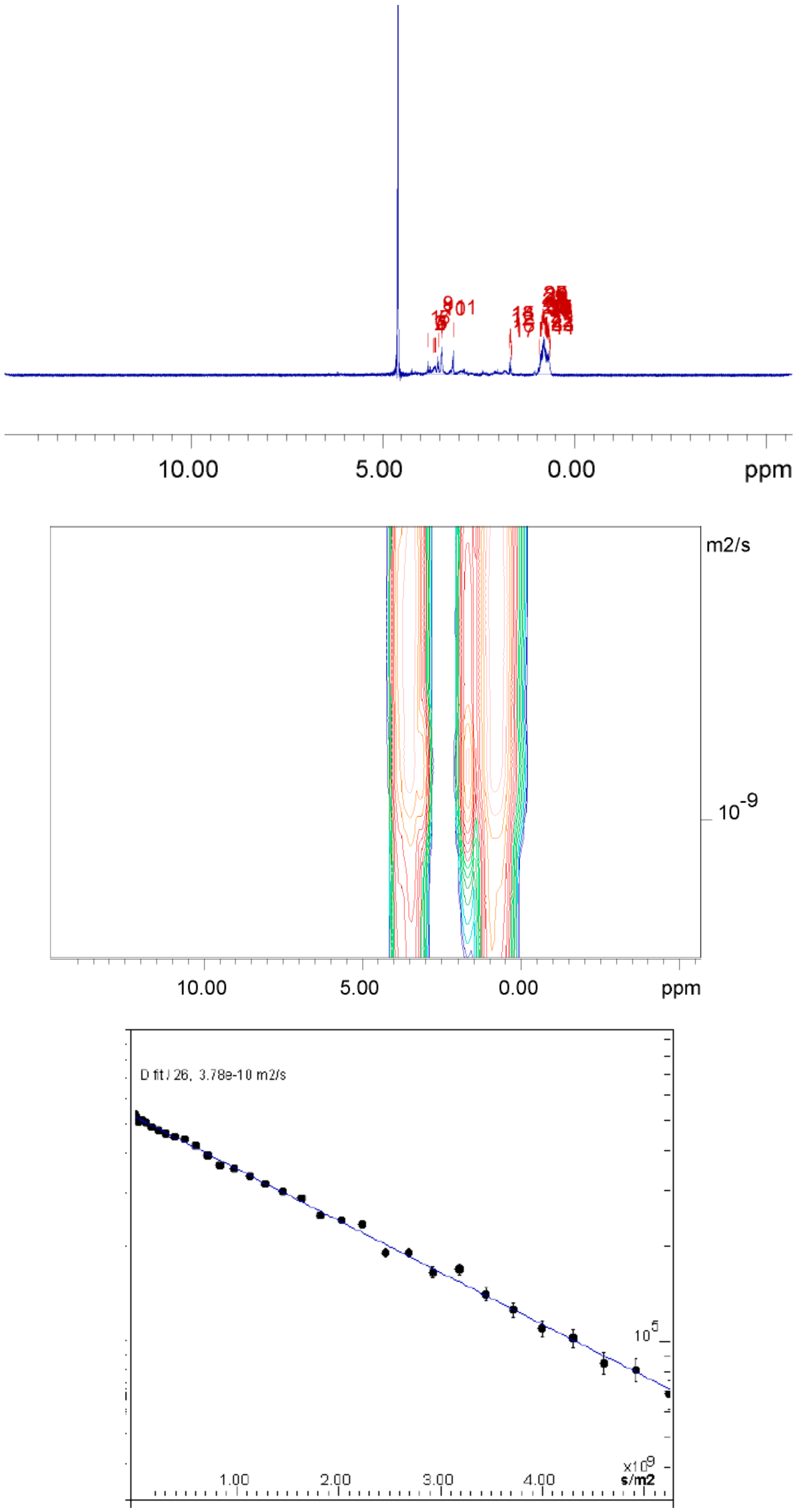

Figure S12: DOSY correlations of **HE-Pep** (400 MHz, D_2O, 298 K) and the attenuation curve.

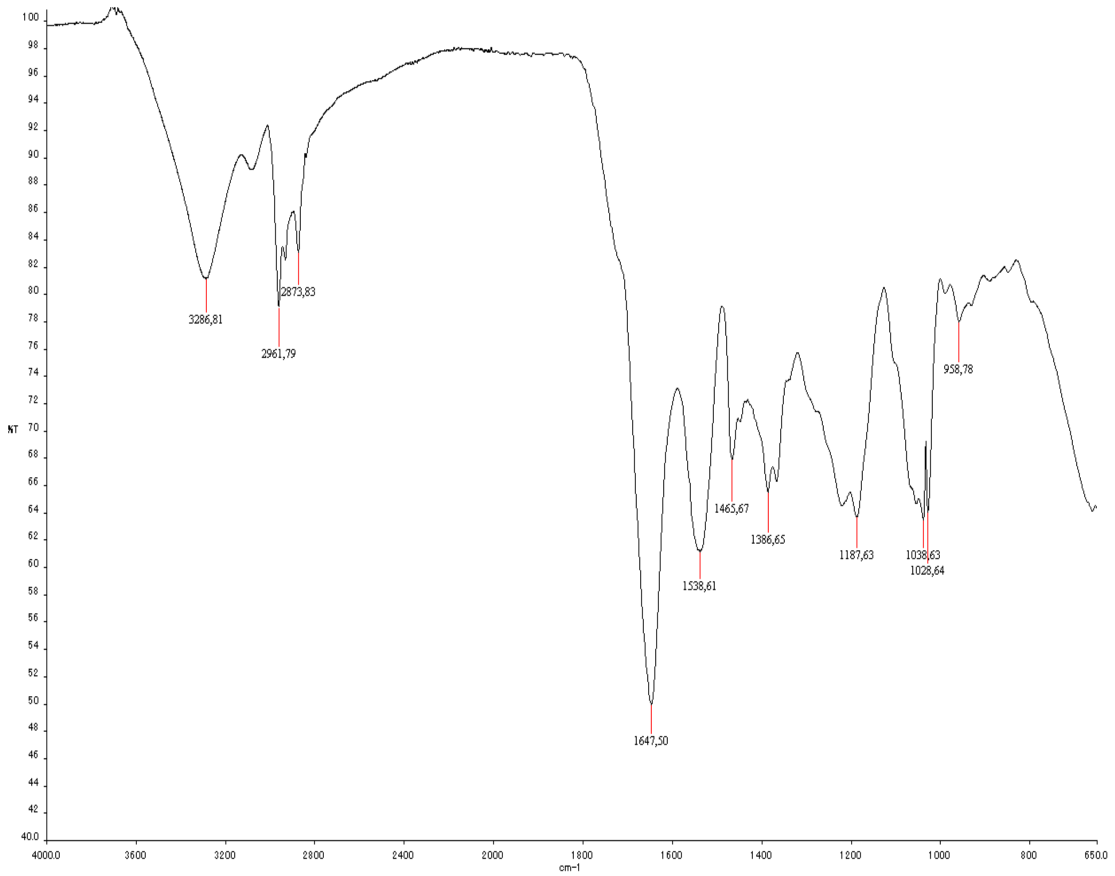

Figure S13: IR spectrum of **HE-Pep** (ATR).

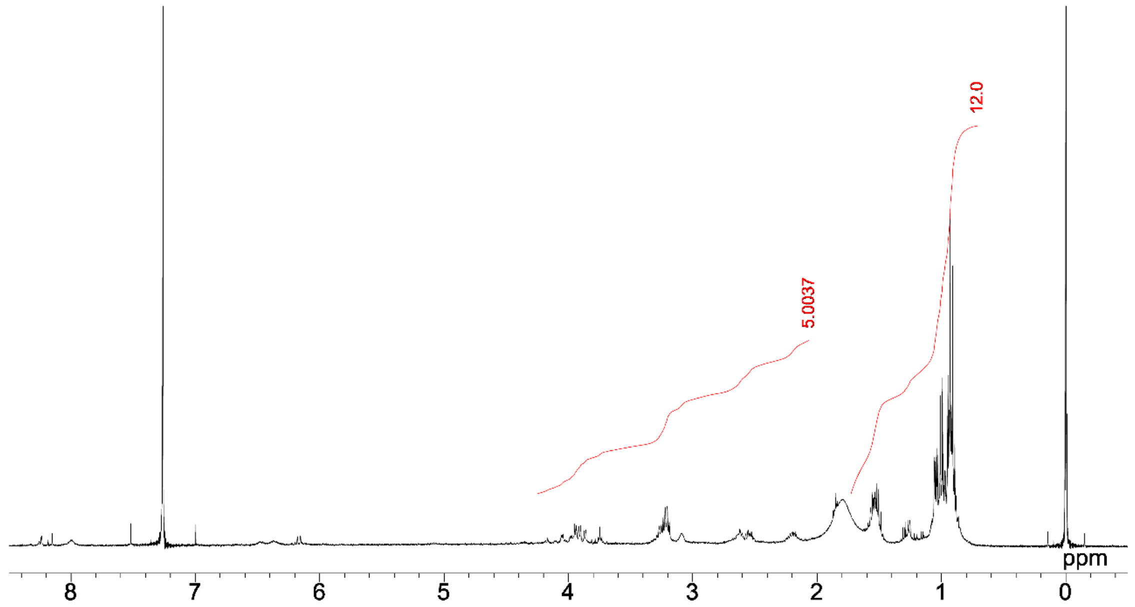

Figure S14: ^{1}H NMR spectrum of **Pr-Pep** (400 MHz, $CDCl_3$, 298 K).

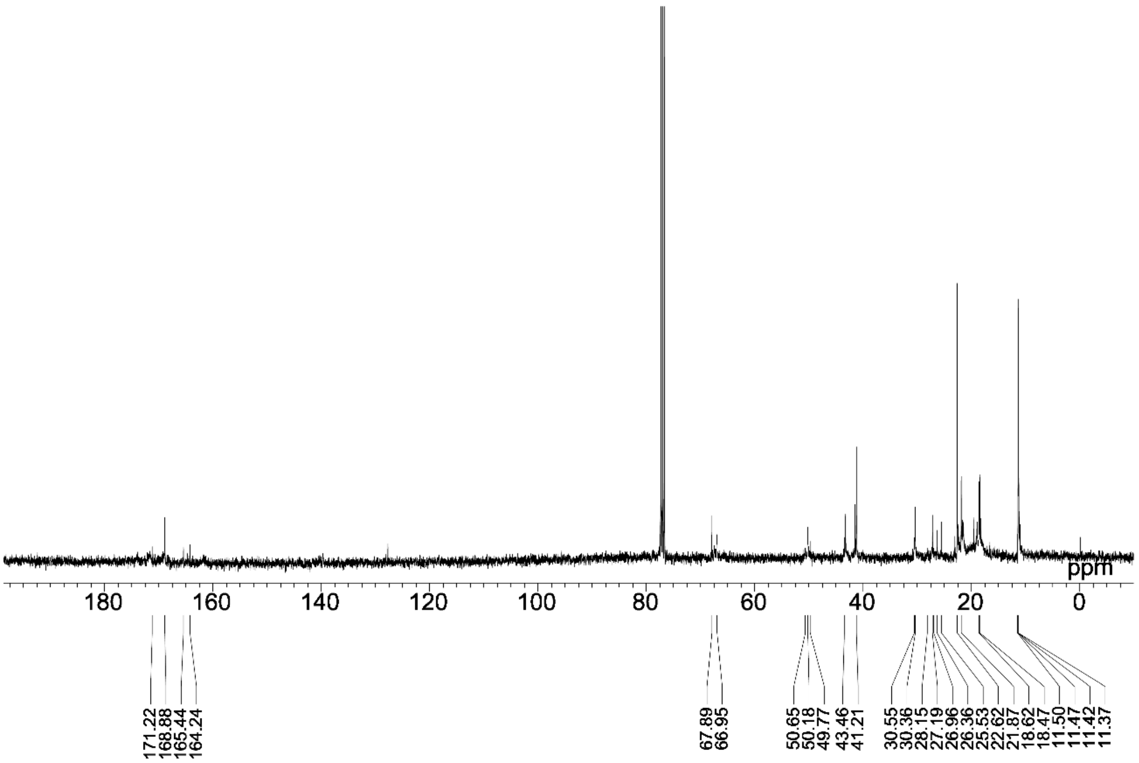

Figure S15: ^{13}C NMR spectrum of **Pr-Pep** (100 MHz, $CDCl_3$, 298 K).

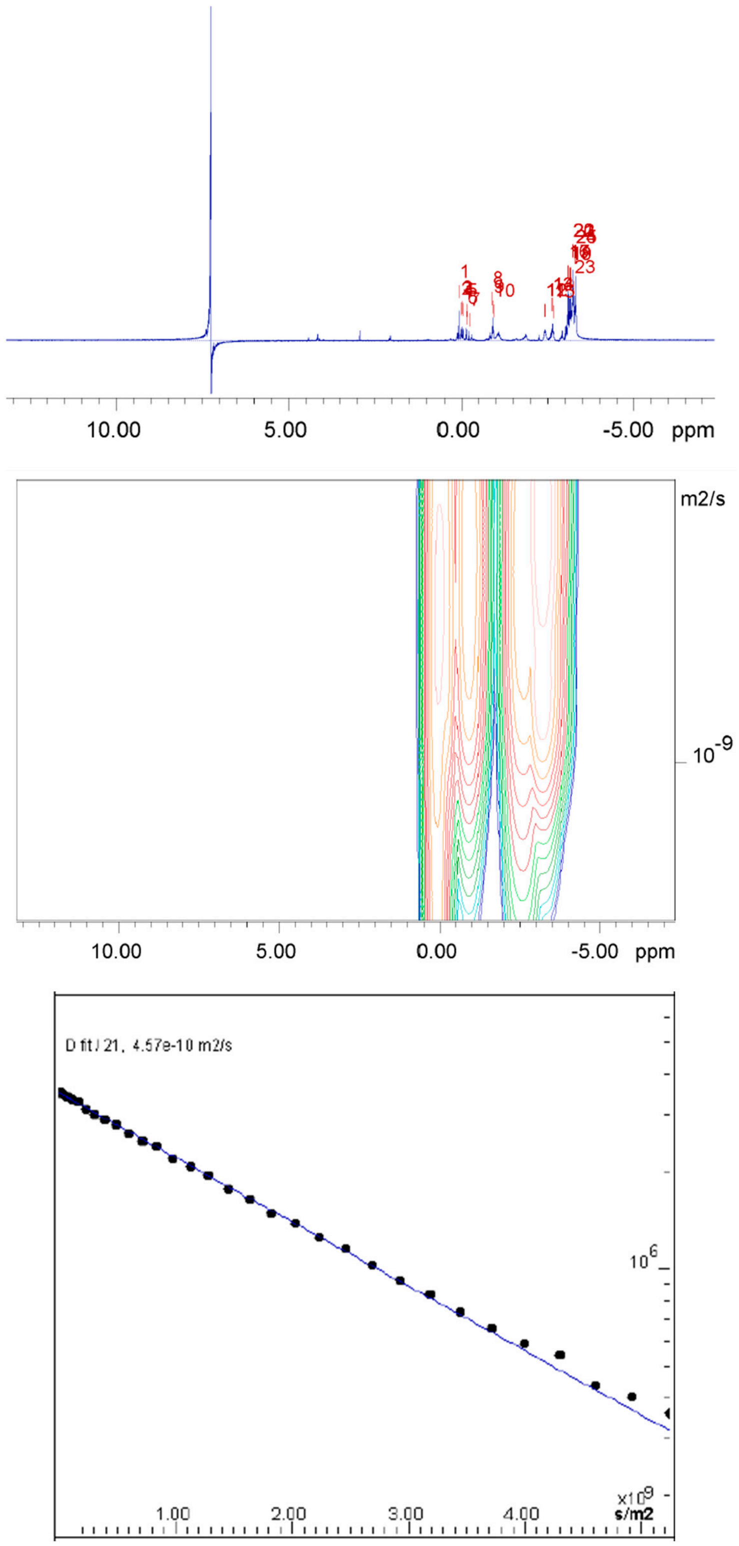

Figure S16: DOSY correlations of **Pr-Pep** (400 MHz, TFA-*d*, 298 K) and the attenuation curve.

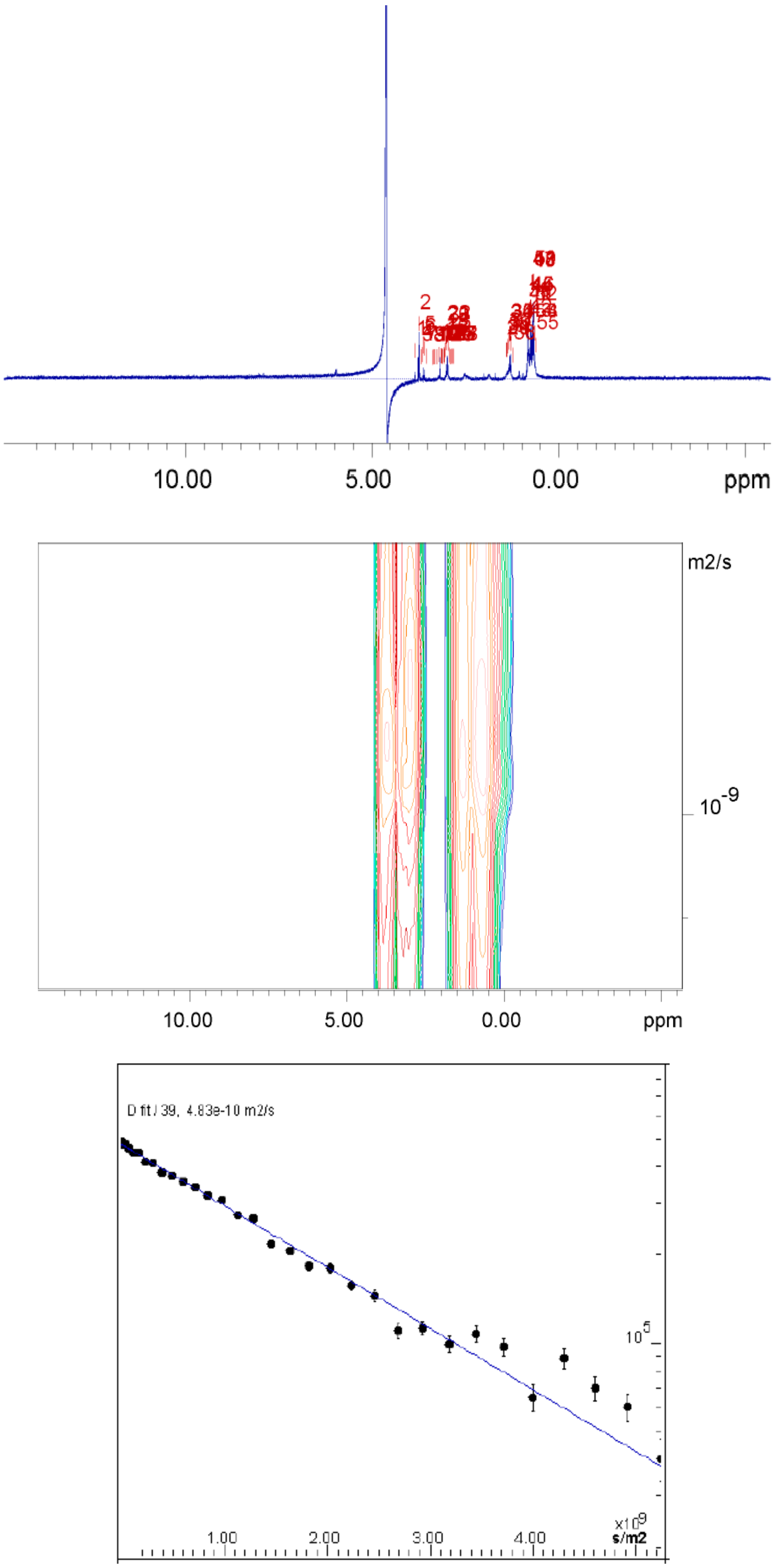

Figure S17: DOSY correlations of **Pr-Pep** (400 MHz, D_2O, 298 K) and the attenuation curve.

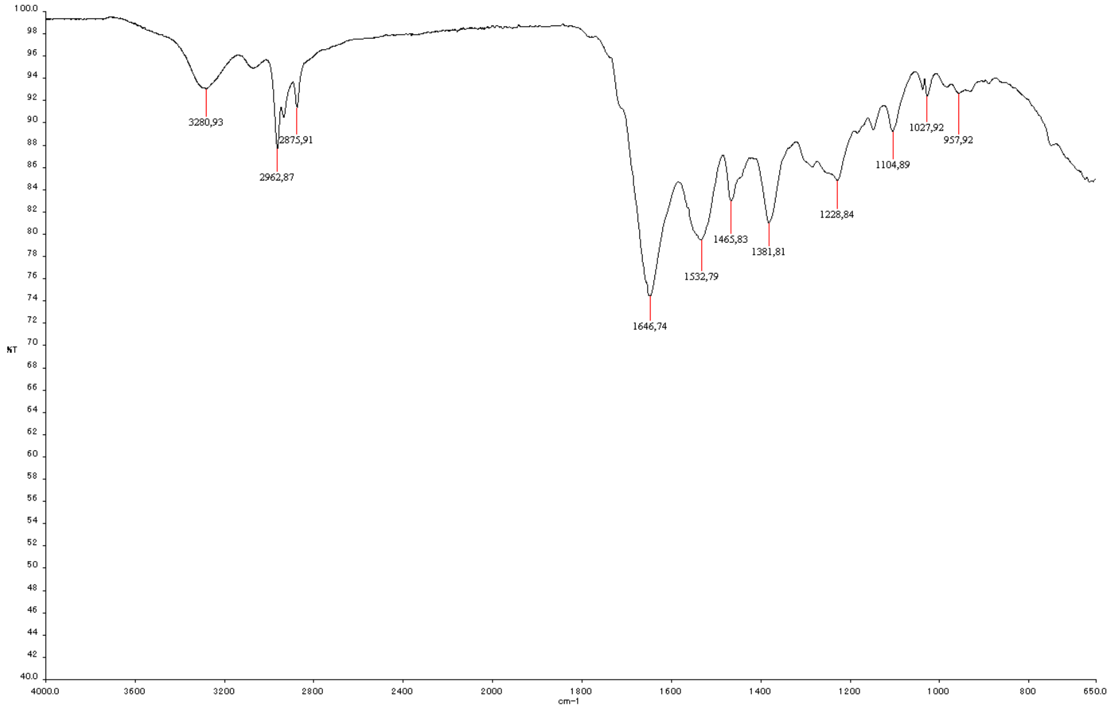

Figure S18: IR spectrum of **Pr-Pep** (ATR).

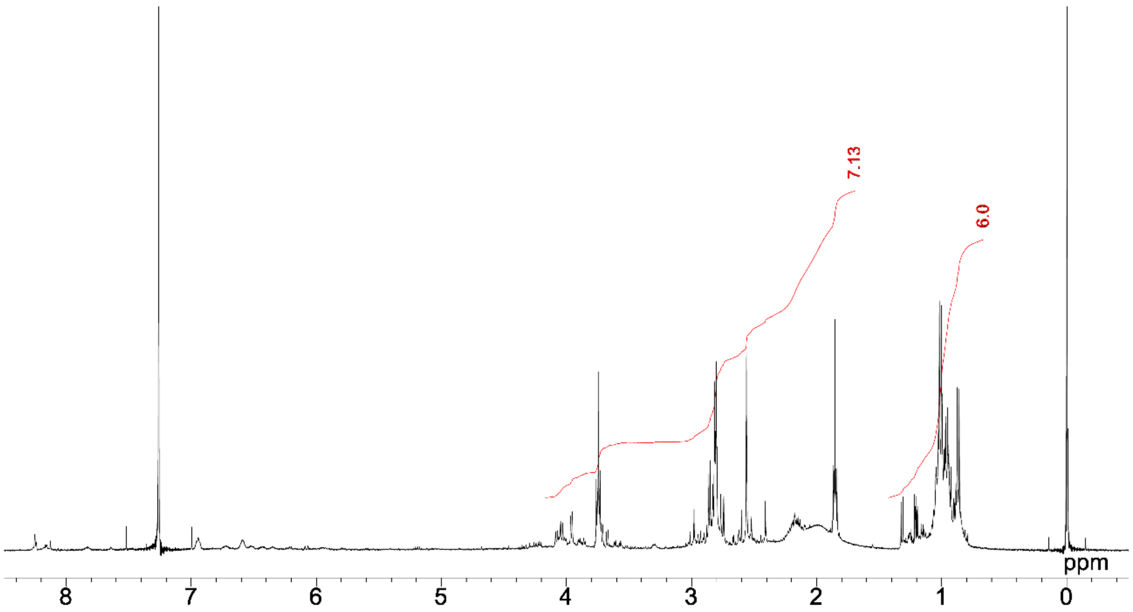

Figure S19: ^{1}H NMR spectrum of **Me-Pep** (400 MHz, $CDCl_3$, 298 K).

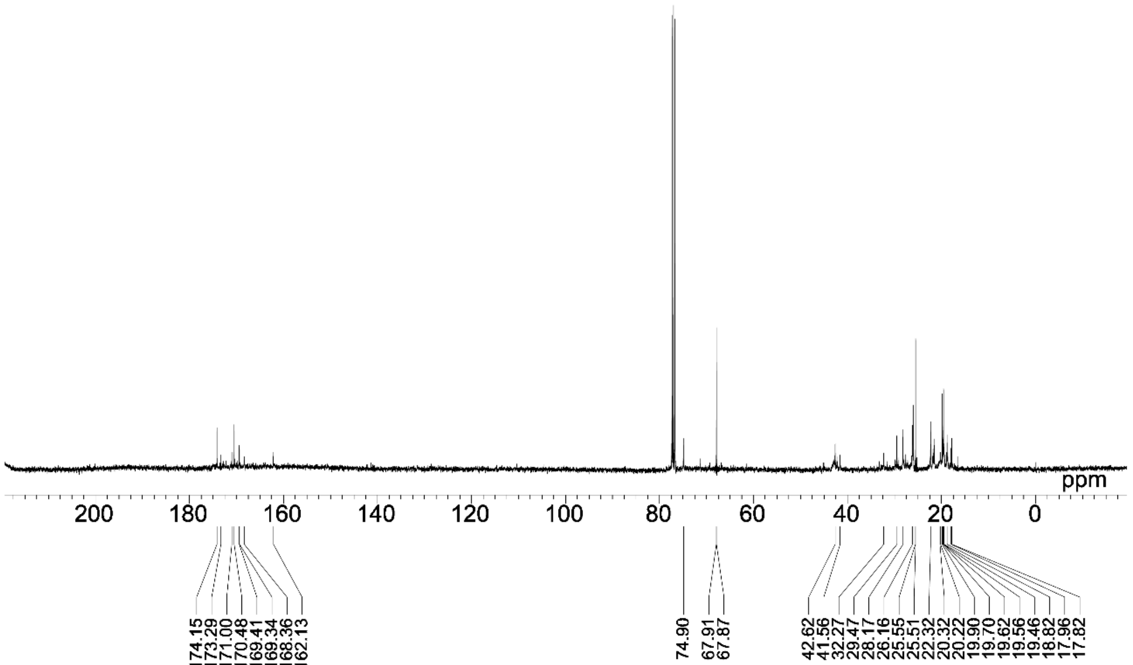

Figure S20: ^{13}C NMR spectrum of **Me-Pep** (100 MHz, $CDCl_3$, 298 K).

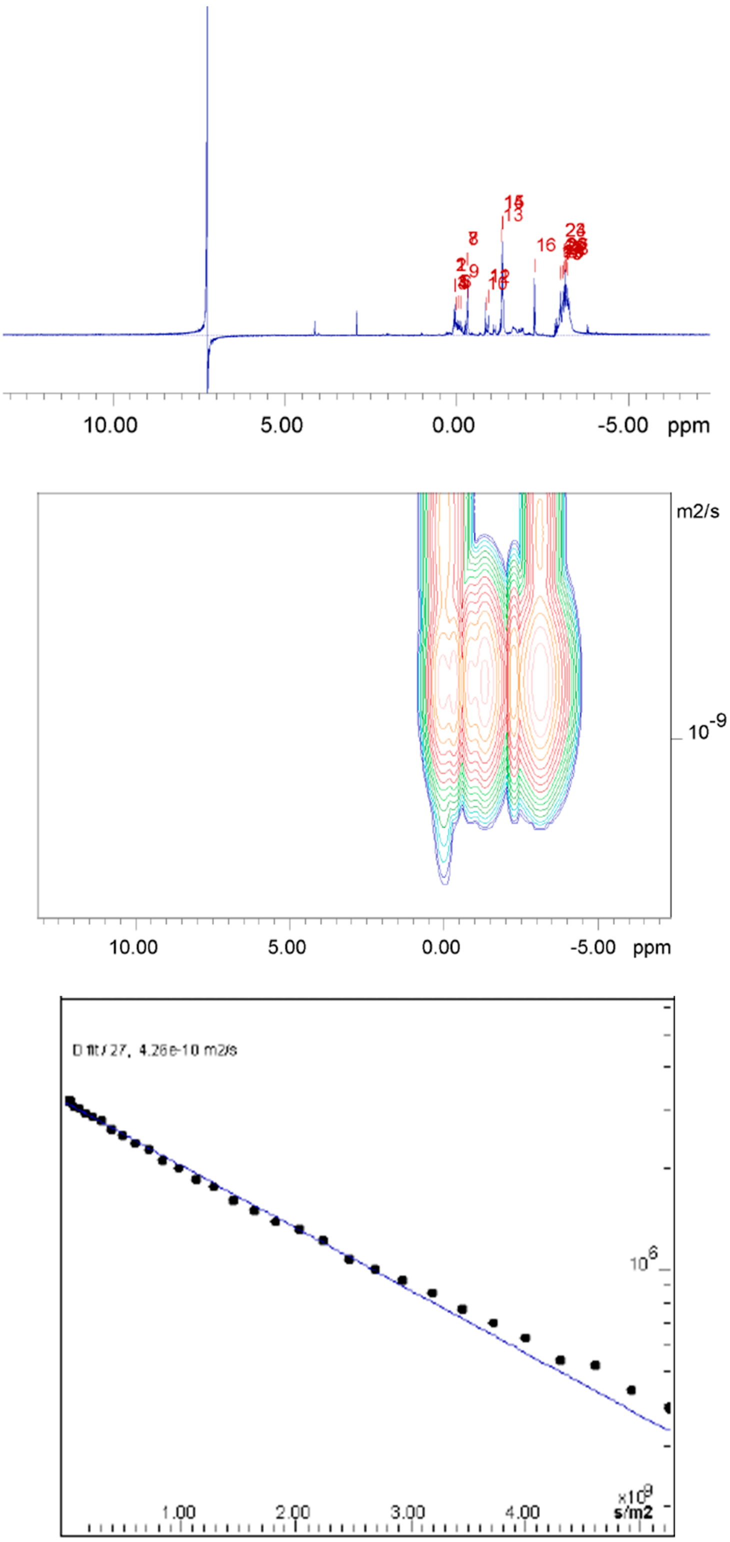

Figure S21: DOSY correlations of **Me-Pep** (400 MHz, TFA-*d*, 298 K) and the attenuation curve.

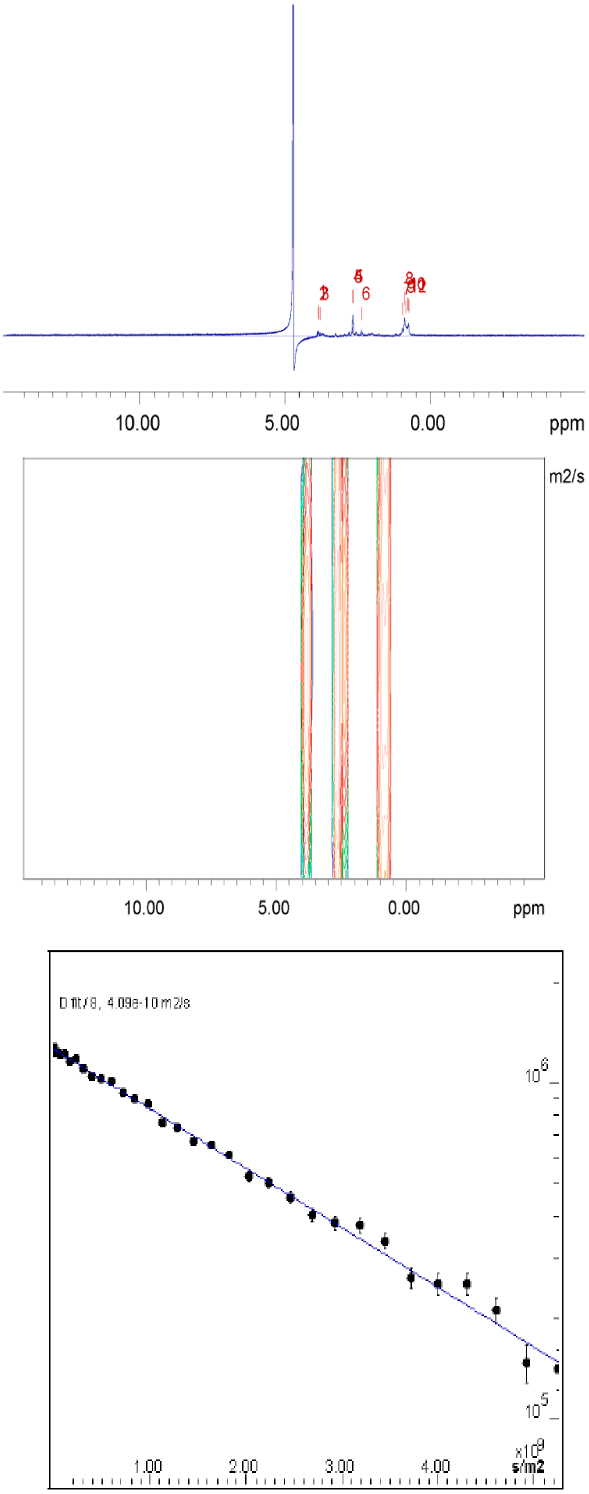

Figure S22: DOSY correlations of **Me-Pep** (400 MHz, D_2O, 298 K) and the attenuation curve.

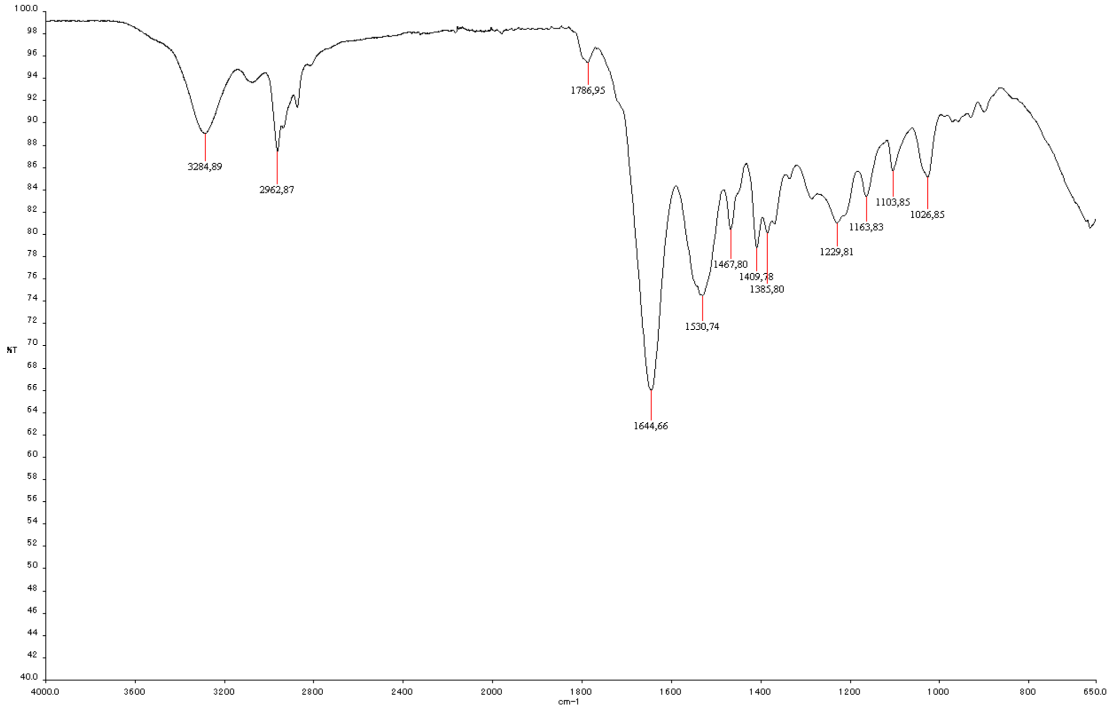

Figure S23: IR spectrum of **Me-Pep** (ATR).

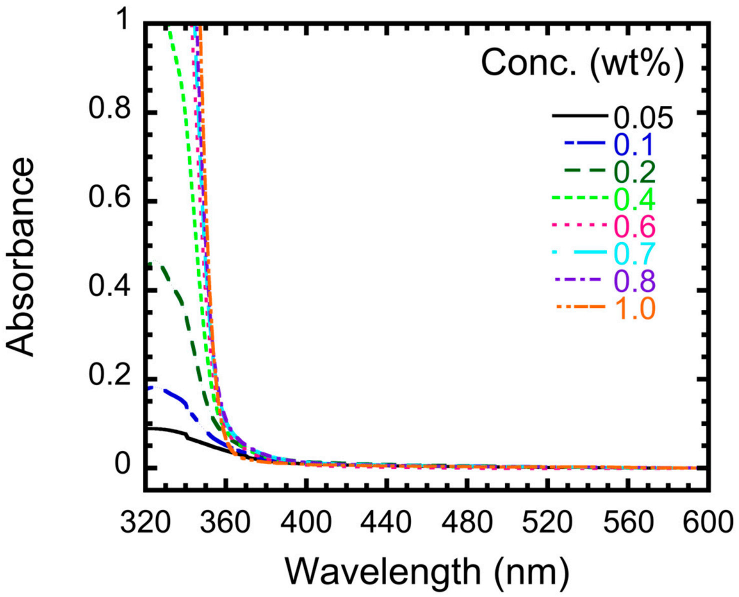

Figure S24. UV-vis spectra of **Poly(Gly-*alter*-L-Val)** in H_2O at various concentrations at 25 °C.

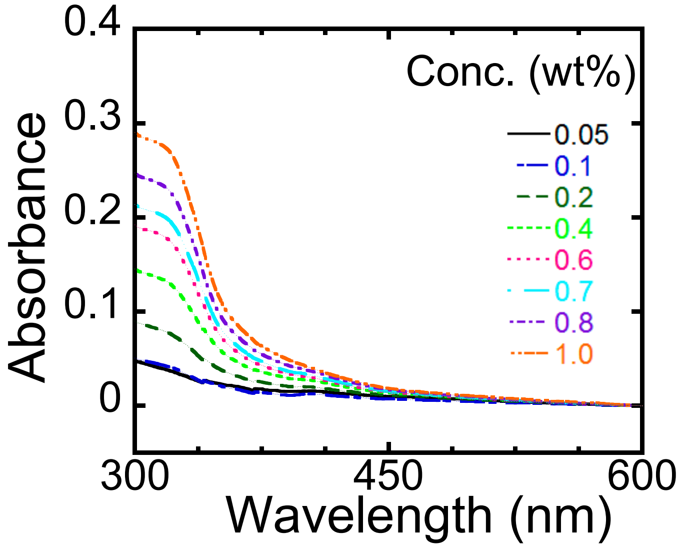

Figure S25. UV-vis spectra of **Et-Pep** in H_2O at various concentrations at 25 °C.

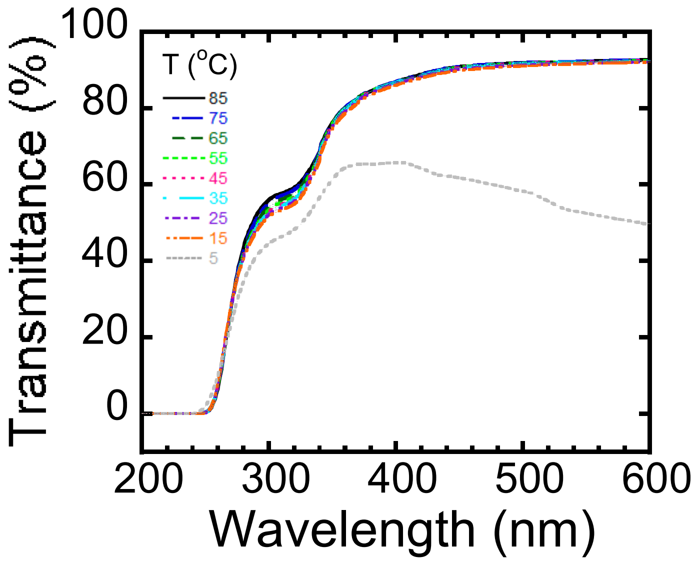

Figure S26. UV-vis spectra of **Et-Pep** in H_2O at various temperatures (1.5 wt%).

Table S1. CMC, cloud point, and hydrodynamic radius (R_h) of **Poly(Gly-*alter*-L-Val)**, **Et-Pep**, and **Me-Pep**.[a]

Alternating Peptide	CMC (wt%)	Cloud point (°C)	R_h in D_2O (R_{water}, nm)	R_h in TFA-*d* (R_{TFA}, nm)	R_{water} / R_{TFA}
**Poly(Gly-*alter*-L-Val)**	0.2	15	0.600 ± 0.031	0.907 ± 0.015	0.66
**Et-Pep**	0.2	10	0.527 ± 0.005	0.556 ± 0.012	0.95
**MePep**	0.2	15	0.538 ± 0.015	0.542 ± 0.010	0.99

[a] Standard deviations are from three samples.
